# Notch Receptor Expression in Neurogenic Regions of the Adult Zebrafish Brain

**DOI:** 10.1371/journal.pone.0073384

**Published:** 2013-09-09

**Authors:** Vanessa de Oliveira-Carlos, Julia Ganz, Stefan Hans, Jan Kaslin, Michael Brand

**Affiliations:** Biotechnology Center and Center for Regenerative Therapies Dresden, Technische Universität Dresden, Dresden, Germany; Instituto de Medicina Molecular, Portugal

## Abstract

The adult zebrash brain has a remarkable constitutive neurogenic capacity. The regulation and maintenance of its adult neurogenic niches are poorly understood. In mammals, Notch signaling is involved in stem cell maintenance both in embryonic and adult CNS. To better understand how Notch signaling is involved in stem cell maintenance during adult neurogenesis in zebrafish we analysed Notch receptor expression in five neurogenic zones of the adult zebrafish brain. Combining proliferation and glial markers we identified several subsets of Notch receptor expressing cells. We found that 90 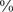 of proliferating radial glia express *notch1a*, *notch1b* and *notch3*. In contrast, the proliferating non-glial populations of the dorsal telencephalon and hypothalamus rarely express *notch3* and about half express *notch1a/1b*. In the non-proliferating radial glia *notch3* is the predominant receptor throughout the brain. In the ventral telencephalon and in the mitotic area of the optic tectum, where cells have neuroepithelial properties, *notch1a/1b/3* are expressed in most proliferating cells. However, in the cerebellar niche, although progenitors also have neuroepithelial properties, only notch1a/1b are expressed in a high number of PCNA 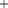 cells. In this region *notch3* expression is mostly in Bergmann glia and at low levels in few PCNA 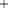 cells. Additionally, we found that in the proliferation zone of the ventral telencephalon, Notch receptors display an apical high to basal low gradient of expression. Notch receptors are also expressed in subpopulations of oligodendrocytes, neurons and endothelial cells. We suggest that the partial regional heterogeneity observed for Notch expression in progenitor cells might be related to the cellular diversity present in each of these neurogenic niches.

## Introduction

Teleost fish, like many non-mammalian vertebrates, display widespread neurogenesis in adulthood (see review(s) [1]–[6]). Several proliferation zones were identified in distinct regions along the rostrocaudal axis, mainly located at the ventricular surfaces [7], [8]. These zones contain precursor cells that actively cycle and generate offspring that migrates out to the mantle zone [8]. This is in contrast to neurogenesis in the adult murine brain, which is restricted to only two zones in the telencephalon – the subventricular zone (SVZ) of the lateral ventricle and the subgranular zone (SGZ) of the dentate gyrus (DG), in the hippocampus – and in the hypothalamus [9]. In mammalian models, these regions have been characterized at the ultrastructural level and their cellular composition and the molecular properties of the different cell types within these niches are known in detail (see review(s) [10]–[12]). Several lines of evidence suggest that some embryonic radial glia cells are neurogenic progenitors/neural stem cells (NSCs), that keep these properties throughout development and give rise to the SVZ cells (see review(s) [13], [14]). However, few of the neurogenic niches have been analysed with respect to their cellular composition in the adult teleost brain [15]–[20].

In the zebrafish dorsal telencephalon, the cellular composition of progenitors is mixed, with a fraction of cells that do not display glia characteristics intermingled with others that show markers and morphology typical of radial glia [16], [18]. In contrast, in the ventral part of the ventral telencephalon [18], optic tectum [17] and cerebellum [15], progenitor cells do not display radial glial properties but rather maintain neuroepithelial-like characteristics. It is still not understood how this divergence in the progenitor properties is achieved and what factors influence it.

The Notch pathway is a conserved pathway throughout the animal kingdom and has been intensely studied for its crucial role in cell fate decision, proliferation and cell death during embryonic neural development (for review see [21]–[26]). In the mammalian brain, both during development and in adulthood, active Notch signaling is required for NSCs maintenance [27]–[30] and self-renewal [29]–[31]. Studies have shown that Notch receptor activation suppresses neuronal [32]–[35] and oligodendrocyte differentiation [33], [34], [36]–[39] while promoting astrogliogenesis [33], [34], [40]. Expression studies in the murine embryonic telencephalon revealed that indeed several Notch receptors are present in the ventricular zone, where progenitors reside [40]–[42]. Also, in the postnatal and adult mouse brain, expression of Notch1 receptor was detected in SVZ astrocytes, in migratory neuroblasts, in the ependymal layer, in a few cells of the olfactory bulb, in the subgranule cell layer of the dentate gyrus, and in Purkinje cells [29], [30], [34], [35], [43], [44]. Some of the Notch expressing cells in the SVZ are proliferating and the expression levels decay with age [34]. In the same study it was observed that Notch activity in SVZ reactive astrocytes is increased upon cortical injury [34].

It has been recently proposed that, in the dorsal telencephalon of adult zebrafish, Notch signaling is responsible for turning neurogenic progenitors quiescent by blocking their proliferative capacity [45]. However, because the lineage relationships between cell types are not clear, the role of Notch signaling in the maintenance of the progenitor niche itself has not been clarified so far. It is not understood whether the different marker expression in the adult dorsal telencephalon [16], [18] reveals distinct precursor populations, or if there is a common multipotent progenitor at the top of the hierarchy. The presence and requirement of Notch signaling at different steps of the progenitor hierarchy or in different progenitor subtypes has not been addressed so far in zebrafish.

Multiple Notch receptors are present in the zebrafish genome, namely *notch1a*, *notch1b*, *notch2* (previously *notch6*) and *notch3* (previously *notch5*) [46], [47], and only *notch2* expression is excluded from the nervous system during embryonic development [47]. Expression of these genes has been observed in the adult zebrafish telencephalon [45].

Because the cellular composition of progenitor cells in the adult zebrafish brain shows great diversity and different responses of progenitors to Notch signaling has been reported in mammals, we analysed in detail the expression of the four Notch receptors in five adult zebrafish neurogenic niches. To this end, we characterized the expression pattern of Notch receptors by *in situ* hybridization and co-stained them with glial, neuronal, oligodendrocytic, endothelial and proliferation markers. In the adult zebrafish brain, we find all known Notch receptors to be expressed, except for *notch2*. Our analysis reveals that *notch1a*, *notch1b* and *notch3* are expressed in all five neurogenic regions of the adult zebrafish brain and that they localize with the majority of the proliferating cells. Similar to embryonic stages, we also find overlapping and complementary expression patterns of Notch receptors in different adult neurogenic niches. The expression of Notch receptors in proliferating cells suggests that Notch signaling might be required at a basal level for maintaining proliferation of neural progenitors.

## Results

We focused our analysis on five neurogenic zones: the dorsal and ventral telencephalon, the hypothalamus, the optic tectum, and the cerebellum. It has been shown that proliferating cells in these areas are periventricularly located [8] or maintain contact with the ventricle by an apical process [15]. Some of these cells are radial-glia [7], [16], [18], [20] but others display a more neuroepithelial-like morphology [15], [17]. We examined if Notch receptors expression differs among niches or if it is conserved and relates with the cellular proliferative status. Expression of *notch1a*, *notch1b* and *notch3* but not *notch2* was detected in the adult brain. Thus *notch2* is not further mentioned.

### *notch1a*, *1b* and *notch3* expression in the adult telencephalon

**Figure 1:**
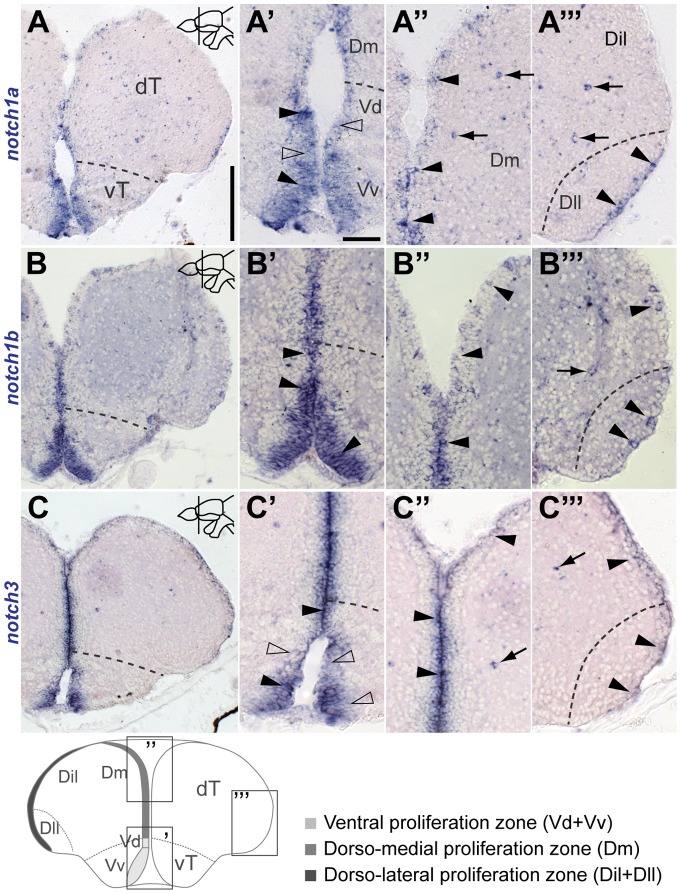
Notch receptor expression in the adult zebrafish telencephalon. Notch receptors are differentially expressed in the ventricular zone of the adult zebrafish telencephalon. Brightfield micrographs of cross-sections at the indicated levels through the telencephalon; the schematic at the bottom-left illustrates the corresponding areas in the cross-section shown at a higher magnification. **A**, **B** and **C**, expression of zebrafish *notch1a*, *notch1b* and *notch3* in the rostral telencephalon, respectively; dashed line indicates the division between ventral and dorsal telencephalon; **A 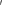
** –**C** 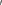, Higher magnification of the ventral expression domain; **A 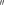
** –**C** 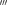, Higher magnification of the dorsal expression domain; dashed line indicates the dorso-lateral most region. Arrowheads and unfilled arrowheads indicate, respectively, strongly and weakly expressing cells located at the ventricular surface; arrows indicate expressing cells in the parenchyme. Abbreviations: Dll, lateral part of the dorso-lateral telencephalic area; Dil, intermediate part of the dorso-lateral telencephalic area; Dm, dorso-medial telencephalic area; dT, dorsal telencephalic area; vT, ventral telencephalic area; Vd, dorsal nucleus of the ventral telencephalon; Vv, ventral nucleus of the ventral telencephalon. Scale bars  = 200 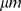 in **A** (applies to **B**, **C**); 25 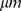 in **A 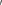
** (applies to **B** 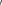,**C** 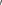, **A 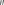
** –**C 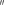
** and **A 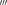
** –**C** 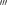).

In the adult zebrafish telencephalon, *notch1a*, *notch1b* and *notch3* are mostly expressed in cells lining the ventricular surface (Fig. 1A–C). In the ventricular zone of the ventral telencephalon (vT) all three are strongly expressed (Fig. 1A 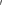 –C 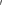). In the dorsal telencephalon (dT), *notch1a* 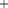 and *notch1b* 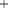 cells are unevenly distributed, with a higher density in the ventricular zone of the dorsal-medial telencephalic area (Dm) (Fig. 1A 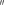, B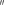) and in small clusters of cells in the ventricular zone of the lateral part of the dorsal-lateral telencephalic area (Dll) (Fig. 1A 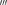, B 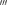). In the ventricular zone of the intermediate part of the dorso-lateral telencephalic area (Dil) a few scattered *notch1a* 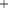 and *notch1b* 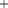 cells are observed (Fig. 1A 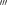, B 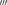). In contrast, *notch3* 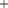 cells are present throughout the whole dT, uniformly distributed both in Dm and Dll (Fig. 1C–C 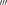). These expression patterns are found along the rostro-caudal axis of the adult telencephalon. Some scattered cells within the parenchyme are positive for *notch1a, notch1b* and *notch3* both in the dT and vT (Fig. 1A, B, C). To assess the identity of these Notch receptor positive parenchymal cells, we preformed double fluorescence *in situ* hybridization (FISH) for the receptors and *olig2*, a marker for oligodendrocytes. This revealed that all three Notch receptors are expressed in a subpopulation of *olig2* 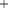 cells (Fig. S1). Using the reporter line *Tg*(*fli*1:gfp) [48], that labels endothelial cells, we found that *notch1a* and *notch3* localize in a subset of these GFP 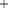 cells (Fig. S2).

### *notch1a*, *1b* and *notch3* expression in radial glia and proliferating cells of the dorsal telencephalon

In the dorsal telencephalon different cell populations have been previously characterized based on their proliferative status and presence of glial markers [18]. To further characterize the cell types that express the Notch receptors, we analysed the co-localization of Notch receptor positive cells with the glial marker S100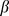 and PCNA, a marker for proliferating cells. To this end, we counted the number of ventricular cells positive for S100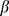, PCNA and Notch receptors in the indicated areas of Dm and Dl (Fig , 3, Table S1).

**Figure 2:**
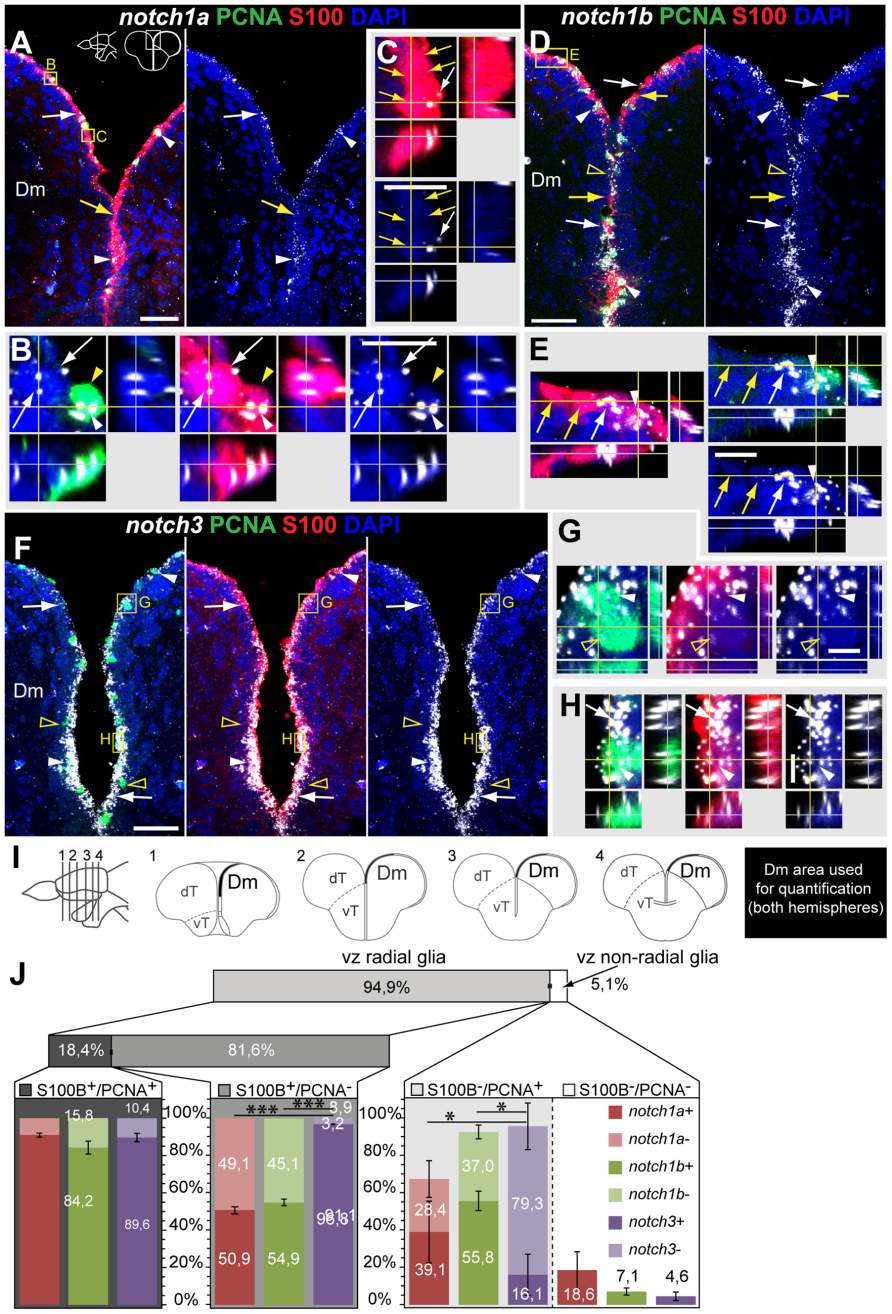
*notch1a*, *notch1b* and *notch3* expression in radial glia and proliferating cells of the dorso-medial ventricular zone of the telencephalon. Confocal images showing localization of Notch receptors by FISH (white), radial glia labelled with S100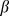 (red), and PCNA 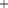 proliferating cells (green); DAPI (blue) is used as nuclear counterstaining. Schematics in A indicate the cross-section levels through the telencephalon and the dorsal telencephalic area represented in the micrographs. **A–C**, *notch1a* and **D–E**, *notch1b* expressing cells are scattered throughout the dorso-medial ventricular zone of the dT and co-localize with both PCNA 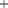/S100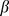 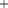 cells (filled arrowheads) and a subpopulation of PCNA 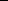/S100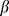 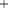 cells (white arrows); yellow arrows indicate Notch receptor 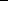 /PCNA 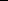/S100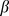 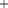 cells; unfilled yellow arrowheads indicate *notch1b* 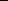 /PCNA 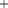 cells. **F–H**, *notch3* expressing cells localize to a great extent with the S100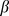 marker, including both PCNA 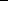 (white arrows) and PCNA 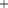 (white arrowheads) cells; unfilled yellow arrowheads indicate *notch3* 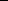 /PCNA 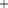 cells. **I**, Schematics indicating the cross-sections levels through the telencephalon, along the rostro-caudal axis, and examples of Dm areas used for marker co-localization analysis in J. **J**, Quantification on the co-localization of Notch receptor by FISH with PCNA and S100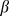, for the Dm region; all ventricularly located cells of the indicated area were counted; cells were distinguished based on their glia character (S100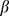 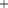), Notch receptor expression and proliferative status (PCNA 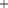). n = 9 (fish), 4–6 tissue sections per fish, at the rostro-caudal levels indicated in I; total number of cells counted = 7245. Values represented as mean percentage 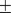 SEM. Significance levels: 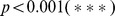, 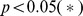. Abbreviations: Dm, dorso-medial telencephalic area; dT, dorsal telencephalic area; vT, ventral telencephalic area; Scale bars  = 50 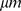 in A, in D and in F; 10 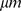 in C (applies to B), in E and in H (applies to G).

**Figure 3:**
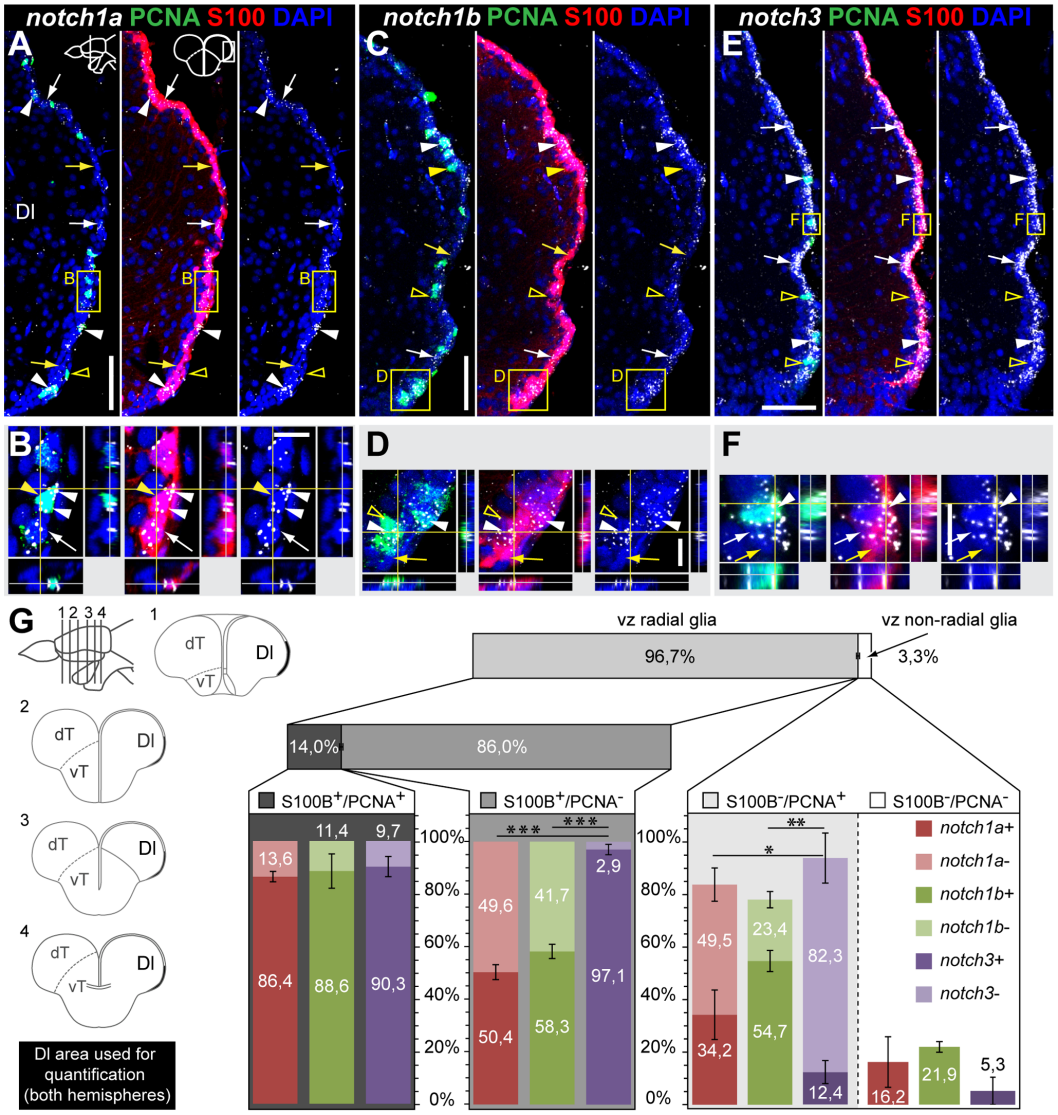
*notch1a*, *notch1b* and *notch3* expression in radial glia and proliferating cells of the dorso-lateral telencephalic ventricular zone. Confocal images showing localization of Notch receptors by FISH (white), radial glia labelled with S100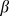 (red), and PCNA 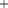 proliferating cells (green); DAPI (blue) is used as nuclear counterstaining. Schematics in A indicate the cross-section levels through the telencephalon and the dorsal telencephalic area represented in the micrographs. **A–B**, *notch1a* and **C–D**, *notch1b* expressing cells are scattered throughout the dorso-lateral ventricular zone of the dT and localize with both PCNA 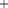/S100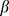 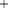 cells (arrowheads) and a subpopulation of PCNA 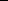/S100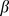 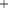 cells (white arrows); yellow arrows indicate Notch receptor 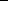 /PCNA 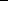/S100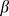 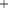 cells; unfilled yellow arrowheads indicate Notch receptor 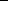 /PCNA 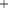/S100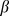 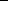 cells. **E–F**, *notch3* expressing cells localize to a great extent with S100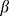, including both PCNA 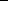 (white arrows) and PCNA 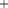 (white arrowheads) cells; unfilled yellow arrowheads indicate *notch3* 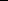 /PCNA 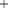 cells. **G**, Quantification on the co-localization of Notch receptor by FISH with PCNA and S100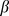, for the Dl region; schematics on the left indicate the cross-section levels through the telencephalon, along the rostro-caudal axis, and examples of Dl areas used for marker co-localization analysis; all ventricularly located cells of the indicated area were counted; cells were distinguished based on their glia character (S100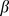 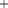), notch expression and proliferative status (PCNA 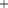). n = 9 (fish), 4–6 tissue sections per fish, at different rostro-caudal levels; total number of cells counted = 6433. Values represented as mean percentage 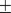 SEM. Significance levels: 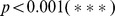, 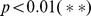, 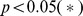. Abbreviations: Dl, dorso-lateral telencephalic area; dT, dorsal telencephalic area; vT, ventral telencephalic area; Scale bars  = 50 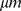 in C (applies to A) and in E; 10 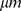 in B, D and F.

**Figure 4:**
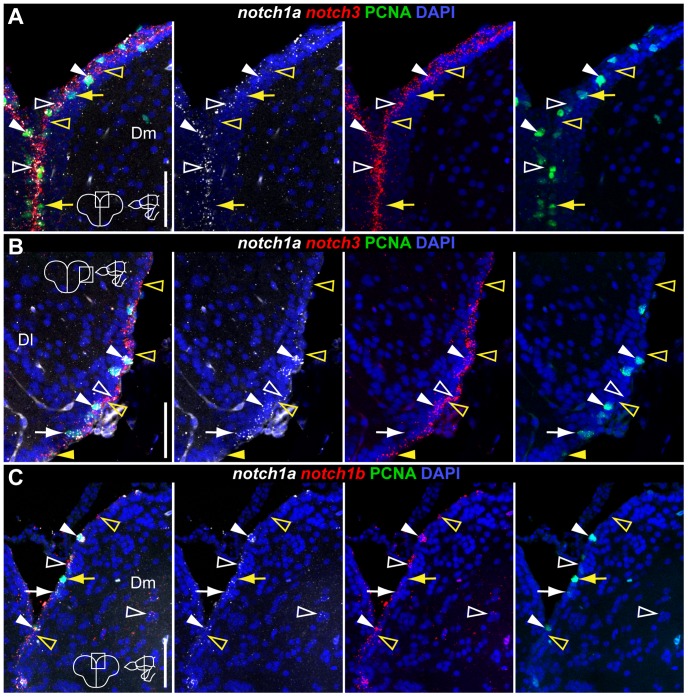
Overlapping and complementary Notch receptor expression in the dorsal telencephalon. Confocal images showing localization of Notch receptors pairs by double FISH (white and red) and PCNA 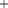 proliferating cells (green); DAPI (blue) is used as nuclear counterstaining. Cross-sections at the indicated level through the telencephalon. Corresponding dorsal telencephalic areas represented in the micrographs are indicated in the cross section schematics of each panel. **A, B**, *notch1a/3* co-expression in Dm and Dl, respectively. **C**, *notch1a/b* co-expression in Dm. These receptors are co-expressed both in PCNA 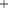 (filled white arrowheads) and PCNA 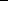 (unfilled white arrowheads) cells; within the PCNA 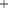 population, few cells are *notch1a* 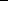 /*notch3* 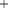 (filled yellow arrowheads) while others are negative for both receptors (filled yellow arrows); within the PCNA 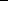 population, some cells express either *notch3* or *notch1b* but not *notch1a* alone (unfilled yellow arrowheads). Scale bars  = 50 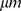.

In agreement with the chromogenic *in situ* hybridization analysis, cells positive for *notch1a* and *notch1b* are scattered along the ventricular surface of the dT (Fig. 2A–B, 3A–B) whereas *notch3* 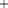 cells are more homogeneously distributed (Fig. 2C, 3C). In Dm, we observed that the majority, 94.9
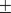
0.4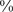 (average
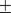
s.e.m.), of ventricular zone cells are S100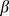 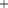, with only 18.4
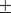
0.3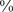 being PCNA 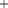 (Fig. 2E). The three Notch receptors are expressed approximately in 90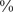 of these proliferating glia cells with no difference observed between them (Fig. 2E). In the PCNA 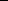 glial cell population, *notch1a* and *notch1b* are present in approximately half of these cells whereas *notch3* expression is found in most of these cells (Fig. 2E). Similar results were obtained for Dl (Fig. 3D). The remaining 5.1
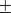
0.4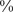 of ventricular zone cells in Dm (and 3.3
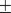
0.4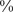 in Dl), do not express the radial glia marker S100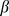 (Fig. 2E, 3D). In this population, most of the proliferating cells do not express *notch3*, while approximately half express *notch1a* and *notch1b* (Fig. 2E, 3D). Moreover, in this ventricular zone S100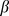 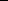 population *notch* 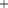 only cells were rare (Fig. 2E, 3D, Table. S1). In summary, *notch3* is expressed in most of the S100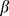 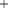 glial cells, irrespective of their proliferative status, whereas *notch1a* and *notch1b* are expressed in a fraction of S100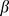 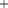 glial cells. These observations lead us to ask if these receptors are co-expressed in the same proliferating cells, distinguishing between a Notch receptor positive and a Notch receptor negative population, or if in combination they cover all proliferating cells. To address this we preformed double FISH for *notch1a/3* and *notch1a/1b*. In agreement with the quantifications above, we observed that, in the dT, *notch1a/3* (Fig. 4A, B) and *notch1a/1b* (Fig. 4C) are co-expressed to a great extent in the PCNA 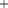 population with only a few proliferating cells expressing either one or the other. Only few proliferating cells are *notch1a* 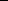 /*3* 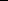 or *notch1a* 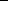 /*1b* 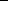. Within the PCNA 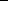 population, *notch1a* 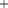 cells are always *notch3* 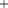, but not vice versa (Fig. 4A, B). Occasionally, PCNA 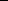 /*notch1a* 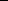 /*notch1b* 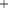 cells can be found (Fig. 4C). This means that only a very small fraction of proliferating cells are negative for Notch receptors.

All together, these results show that i) all three Notch receptors are expressed in approximately the same number of proliferating glial cells, ii) *notch3* is expressed in the majority of S100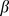 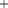 /PCNA 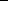 glial cells, whereas *notch1a* and *notch1b* are found just in half of this population, iii) *notch1a* and *notch1b* are often co-expressed in proliferating cells, iv) *notch3* is expressed in very few S100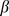 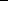 /PCNA 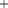 cells in contrast to *notch1a* and *notch1b* which are present in roughly more than half of this population.

### *notch1a*, *1b* and *notch3* show an apical to basal gradient of expression in proliferating cells of the ventral telencephalon

**Figure 5:**
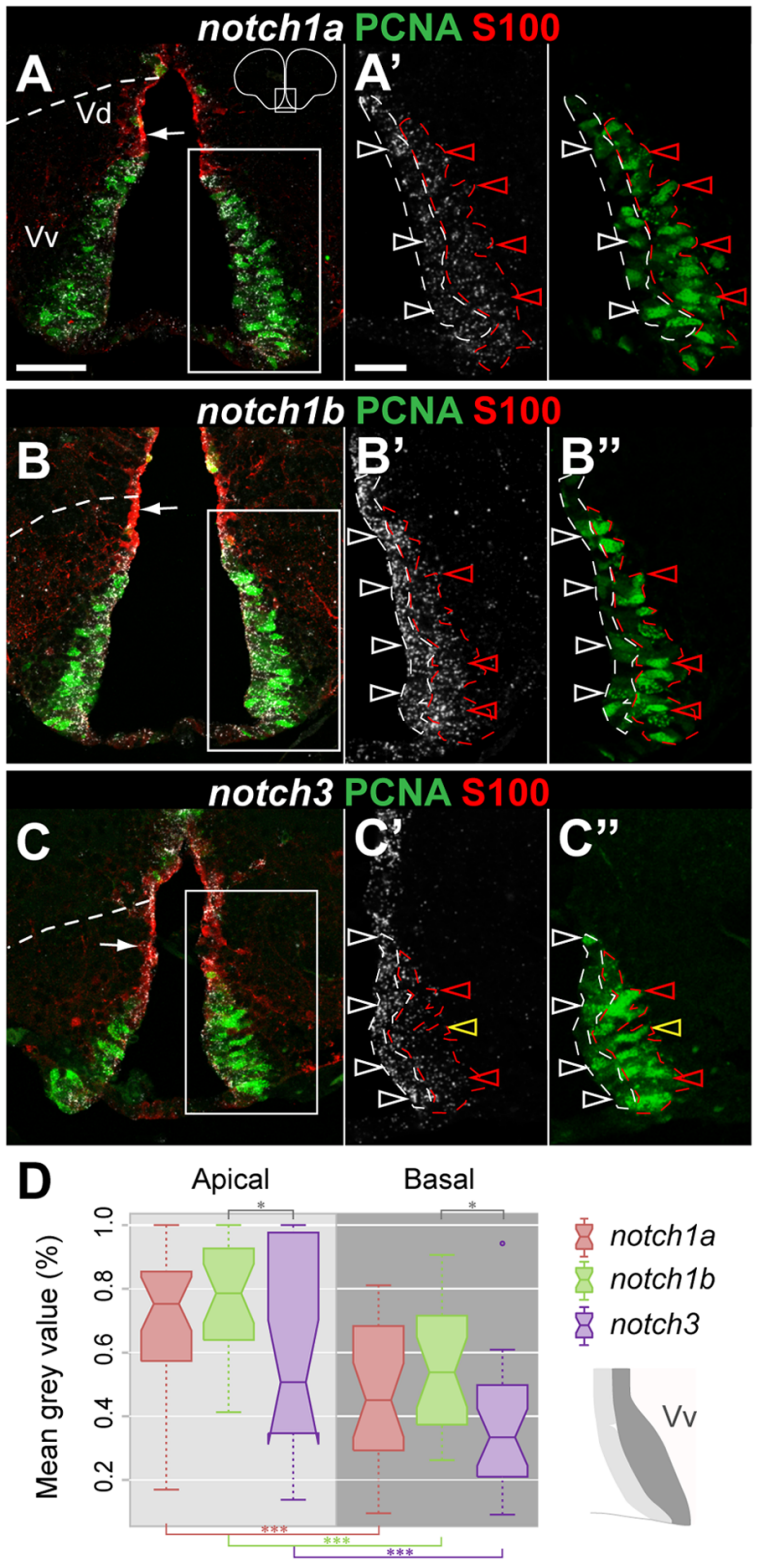
Notch receptor expression in the ventral telencephalic proliferation zone. Confocal images showing the localization of Notch receptors by FISH (white) in PCNA 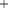 proliferating cells (green) and in the S100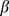 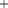 glial cells of Vd (red). **A–A** 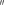, *notch1a*, **B–B
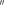
**, *notch1b* and **C–C** 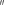, *notch3* expression in the ventral telencephalon. All three receptors are express in S100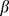 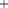 cells of Vd (white arrows). **A 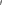 -A** 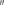, **B 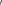 -B 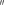
** and **C 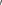 -C 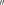
** show the individual channels for Notch receptor expression and PCNA at a higher magnification of the respective boxed areas. Notch receptor 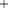 /PCNA 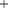 cells with apical nucleus (unfilled white arrowheads) display a stronger expression intensity than PCNA 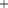 cells with basal nucleus (unfilled red arrowheads); unfilled yellow arrowhead indicate Notch receptor 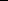 /PCNA 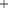 cells. Scale bars  = 50 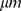 in A (applies to B and C) and 25 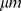 in A 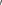 (applies to B 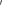, C 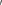 and A 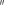 -C 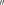). **D**, The notched boxplot represents the relative mean grey values of *notch* expression in proliferating cells with apical or basal nucleus, as illustrated in the schematics and by the respective dashed lines indicated in the higher magnification pictures shown above; the middle line of the box represents the median; the bottom and top of the box represents the 25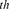 and 75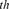 percentiles, respectively; whiskers indicate the minimum and maximum; circle indicates an outlier; total n = 13. Significance levels: 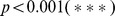, 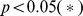.

The ventral telencephalic neurogenic niche is subdivided into the dorsal nucleus of the ventral telencephalon (Vd) and the ventral nucleus of the ventral telencephalon (Vv) [49]. In the proliferation zone of Vd, glial markers are present whereas the proliferation zone of Vv is S100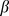 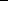 and shows only weak expression of other glial markers [18]. Moreover, the proliferation zone of Vv is positive for the intermediate filament marker Nestin, cells display neuroepithelial-like characteristics and show interkinetic nuclear migration [18]. In Vd, *notch1a* and *notch1b* are expressed in some, but not all of the S100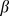 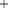 glial cells (Fig. 5A, B), while *notch3* is found throughout the ventricular zone of Vd (Fig. 5C).

**Figure 6:**
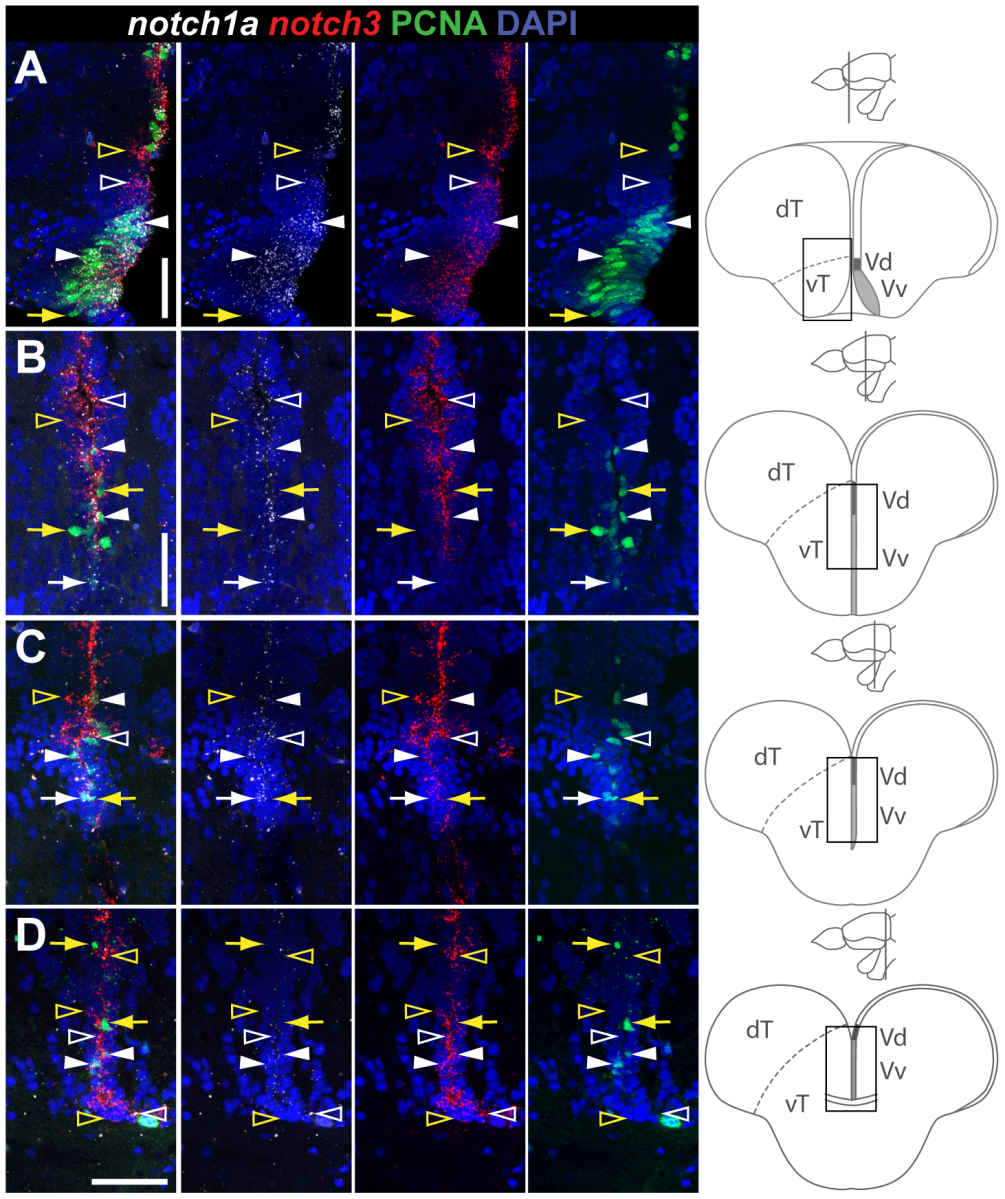
Overlapping and complementary *notch1a/3* expression in the ventral telencephalic proliferation zone, along the rostro-caudal axis. Confocal images showing localization of *notch1a* (white) and *notch3* (red) by double FISH (white and red) and PCNA 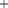 proliferating cells (green); DAPI (blue) is used as nuclear counterstaining. Areas represented in the micrographs are indicated in the cross-section schematics on the right as well as the different levels through the telencephalon. **A–D**, *notch1a* and *notch3* expression domains along the rostro-caudal vT proliferation zone. Co-expression of these receptors both in PCNA 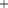 (white filled arrowheads) and PCNA 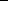 cells (unfilled white arrowheads); a few PCNA 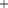 cells are Notch receptor 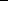 (yellow arrows) while others are *notch1a* 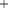 /*notch3* 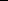 (white filled arrows); unfilled yellow arrowheads indicate *notch1a* 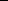 /*notch3* 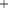 /PCNA 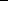 cells. Abbreviations: dT, dorsal telencephalic area; vT, ventral telencephalic area; Vd, dorsal nucleus of the ventral telencephalon; Vv, ventral nucleus of the ventral telencephalon. Scale bars  = 50 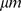 in A, B (applies to C) and D.

In Vv, we observe that Notch receptor expression overlaps with the majority of proliferating cells (Fig. 5), with only very few Notch receptor negative (more obvious caudally) (Fig. 6). Proliferating cells with nuclei either in an apical or basal position show Notch receptor expression. However, expression intensity differs along the apical-basal axis of the proliferation zone. To assess whether there is a differential expression between proliferating cells with distinct apical or basal nuclei positions we divided proliferating cells into two areas (apical and basal) and measured the mean grey value (MGV) for the corresponding areas (for details see Materials and Methods). We found a clear decrease of the fluorescence signal intensity in basally compared to apically located nuclei of proliferating cells. The maximum MGV was always obtained in apical nuclei for all three receptors (Fig. 5D). Moreover, fluorescence profile analysis shows that this gradient can be steeper for *notch3* than for *notch1a/1b* (Fig. S3).These results suggest that Notch receptor expression levels vary during cell cycle.

In contrast to dT, expression of *notch1a*, *notch1b* and *notch3* is very similar in the proliferation zone of vT. To test the overlap of receptor expression in the vT proliferation zone, we performed double FISH for *notch1a* and *notch3* and analysed their expression along the rostral-caudal axis (Fig. 6). At more rostral levels, the majority of proliferating cells express both receptors (Fig. 6A). We also observed that PCNA 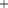 /Notch receptor 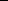 cells are usually basally located and in the most ventral part of Vv (Fig. 6A,. S3B). More caudally, where the telencephalic ventricle closes, the vT proliferation zone is reduced in size [16], [18]. We observed that Notch receptor expression follows this pattern (Fig. 6B–D). Here, we also found PCNA 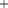 that were either positive or negative for Notch receptor expression. *notch1a* and *notch3* partially overlap in PCNA 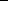 cells that flank the proliferating zone (Fig. 6B–D). However, in this case *notch3* expression is clearly broader than *notch1a*. Occasionally, we found PCNA 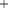 cells expressing only *notch1a* (Fig. 6B).

**Figure 7:**
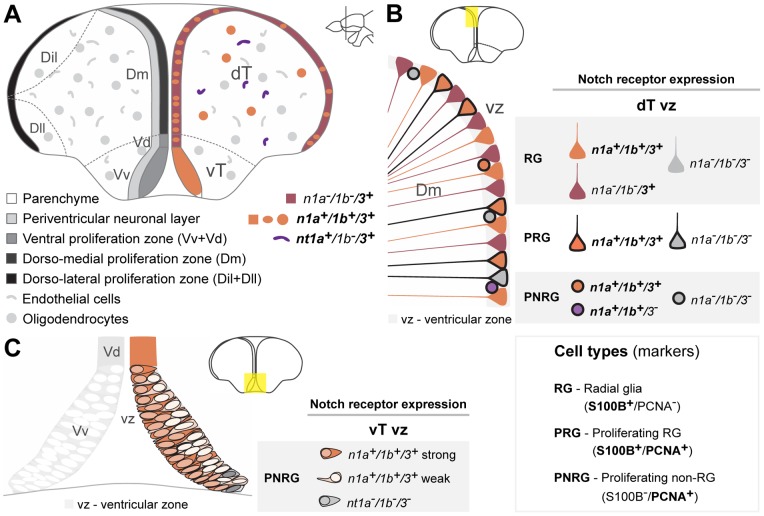
Summary of Notch receptor expression pattern and cellular characteristics in the adult zebrafish telencephalon. **A**, Illustration shows the overall expression of the analysed notch receptors; they are mainly present in ventricular zone cells, with a few positive cells in the parenchyma, overlapping with oligodendrocytic or endothelial markers. **B**, Cellular characteristics of Notch expressing cells in the Dm ventricular zone (vz) and **C**, in the vT proliferation zone. Abbreviations: Dil, intermediate part of the dorso-lateral telencephalic area; Dll, lateral part of the dorso-lateral telencephalic area; Dm, dorso-medial telencephalic area; dT, dorsal telencephalic area; vT, ventral telencephalic area; Vd, dorsal nucleus of the ventral telencephalon; Vv, ventral nucleus of the ventral telencephalon.

In summary, *notch3* shows an overall broad expression compared to *notch1a* and *notch1b* expression, that is present in clusters within the *notch3* 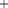 domain (Fig. 7A, B). Notch receptor expressing cells are mainly located along the ventricular zone of the ventral and dorsal telencephalon in PCNA 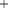 and PCNA 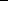 radial glia (Fig. 7). In the rostral vT proliferation zone Notch receptors are very similarly expressed with a stronger signal in cells with apically located nuclei (Fig. 7C).

### *notch1a*, *1b* and *notch3* expression in proliferative areas and radial glia of the adult hypothalamus

The zebrafish hypothalamus is the largest diencephalic area and is subdivided in ventral, dorsal and caudal parts [50]. The hypothalamic proliferation zone is contiguous along these areas and proliferating cells are generally ventricularly located [8]. At the posterior recess in the caudal hypothalamus, proliferation is found periventricularly [8]. S100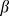 cells are also present in these hypothalamic areas, similar to what has been observed for other teleosts [51], and they are arranged in several adjacent cell layers, extending processes to the ventricular surface (Fig. S4).

**Figure 8:**
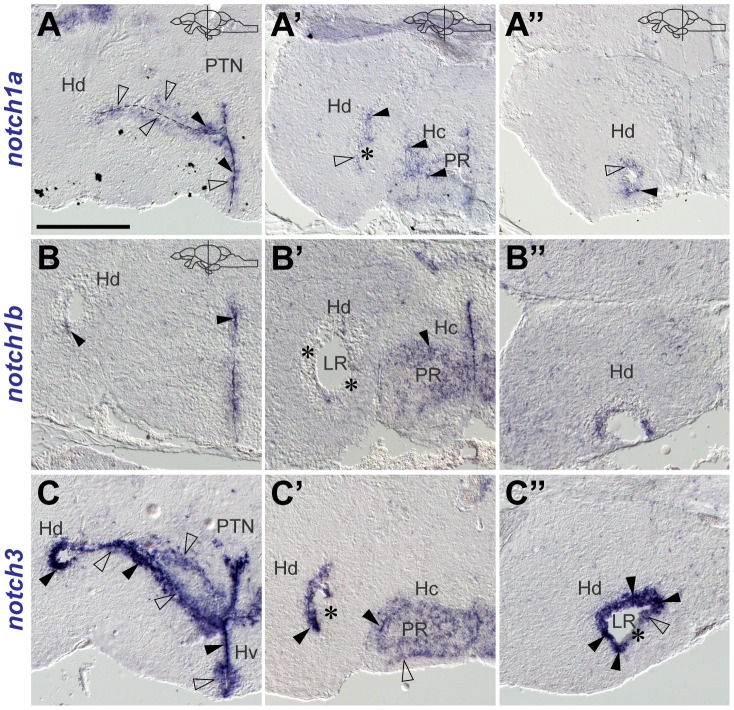
Notch receptor expression in the adult zebrafish hypothalamus. Notch receptors are differentially expressed throughout the hypothalamus. Cross-sections at the indicated level through the diencephalon. Brightfield images show the expression of **A–A** 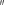, *notch1a*, **B–B** 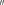, *notch1b* and **C–C** 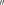, *notch3* at different rostro-caudal levels of the hypothalamus. Strong (filled arrowheads) and weak (unfilled arrowheads) expressing cells are detected. Asterisks (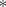) indicate areas of no or very weak expression in the Hd. Abbreviations: DiV, diencephalic ventricle; Hc, periventricular caudal hypothalamus; Hd, periventricular dorsal hypothalamus; Hv, periventricular ventral hypothalamus; LR, lateral recess of the DiV; PR, posterior recess of the DiV; PTN, posterior tuberal nucleus. Scale bar  = 100 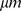 in A (applies to all).

*notch1a* and *notch1b* expression is found along the periventricular ventral hypothalamus (Hv) in groups of cells with different expression intensities (Fig. 8A, B). In Hv, *notch3* expression is continuous and sometimes present a few cells away from the ventricular surface (Fig. 8C).

In the dorsal periventricular hypothalamus (Hd), we detected single scattered *notch1a* 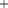 (Fig. 8A 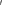, A 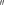) and *notch1b* 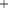 (Fig. 8B 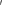, B 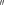) cells at the lateral recess (LR), with different signal intensities. In the LR, most cells did not show *notch1a* or *notch1b* expression (asterisk in Fig. 8A 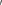 -B 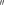, S5B). At the junction between Hv and Hd, additional rows of *notch3* expressing cells are found away from the ventricle (Fig. 8C). Moreover, in Hd/LR *notch3* is strongly expressed in the cells lining the ventricle with a ventral gap in expression that becomes more evident caudally (Fig. 8C–C 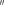).

In the caudal hypothalamus (Hc), at the level of the posterior recess, positive cells for *notch1a* and *notch1b* are arranged in a pattern that resembles the distribution of proliferating cells in this zone as previously shown [8]. In Hc, *notch3* is expressed in a very similar fashion to *notch1b* (Fig. 8C–C 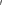).

Co-labelling with the glial marker S100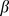 and proliferation marker PCNA revealed that *notch3* covers most of the S100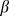 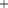 domain and localizes with the majority of S100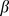 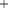 /PCNA 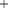 cells (Fig. S4). *notch3* is also expressed in most of the S100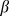 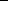 /PCNA 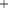 cells, mainly in Hv (Fig. S4). In Hd, PCNA 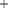 cells are often *notch3* 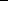 (Fig. S4C, F). Double FISH for *notch1a/3* shows that *notch1a* is expressed in part of the *notch3* domain (Fig. S5). We observed that few proliferating cells are negative for both receptors (Fig. S5).

**Figure 9:**
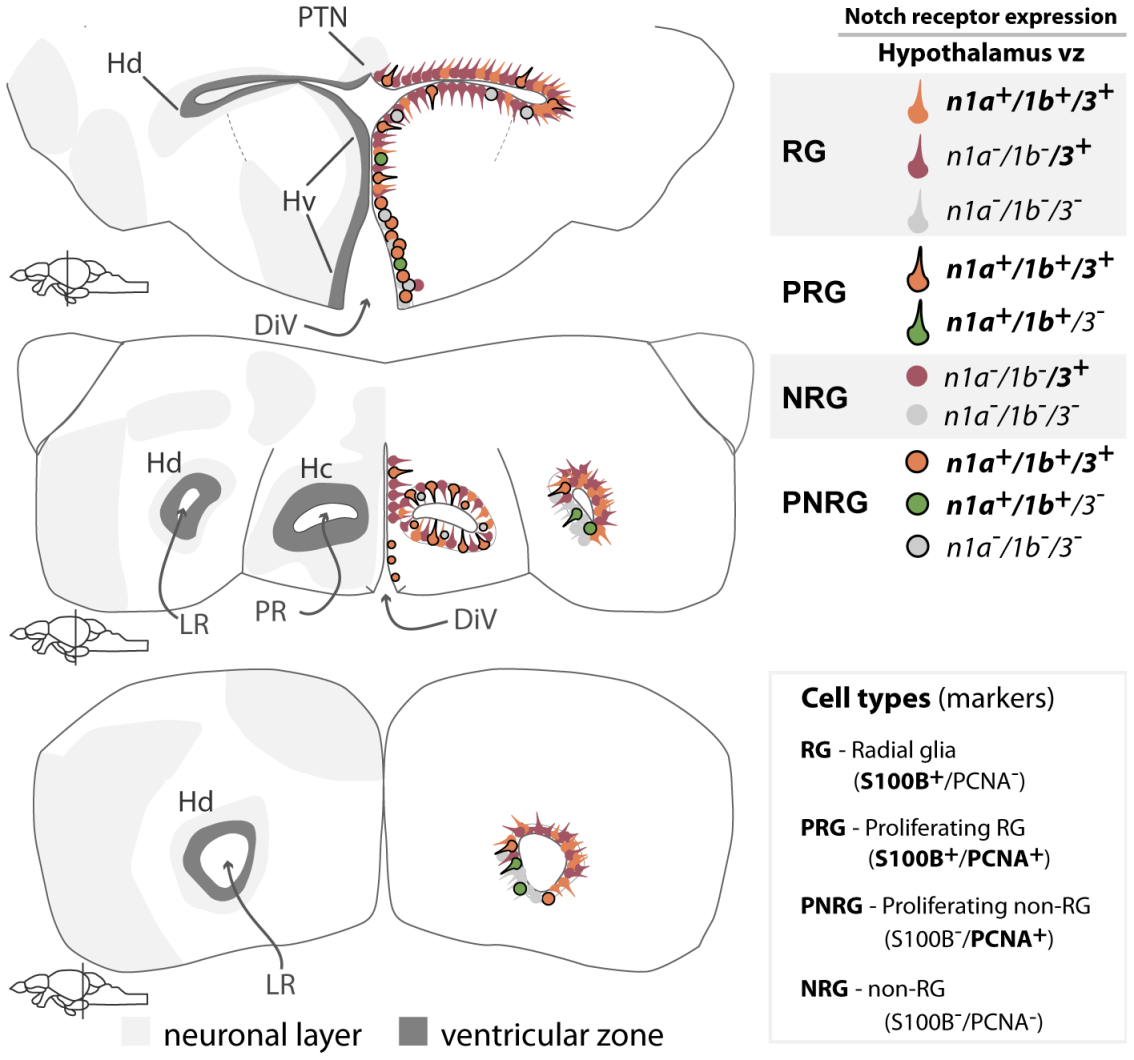
Summary of Notch receptor expression pattern and cellular characteristics in the adult zebrafish hypothalamus. Illustration shows the overall expression of the analysed Notch receptors at different rostro-caudal levels of the hypothalamus; they are mainly present in ventricular zone cells of Hv, Hd and Hc and localize with glial and proliferation markers. Abbreviations: DiV, diencephalic ventricle; Hc, periventricular caudal hypothalamus; Hd, periventricular dorsal hypothalamus; Hv, periventricular ventral hypothalamus; LR, lateral recess of the DiV; PR, posterior recess of the DiV; PTN, posterior tuberal nucleus.

In summary, Notch receptors are expressed in the hypothalamic periventricular areas, partially co-localizing with proliferating cells (Fig. 9). *notch3* expression is continuous throughout the ventricular zone, and along the rostro-caudal axis, with the exception of the LR, where it partially excludes proliferating cells (Fig. 9). *notch1a* and *notch1b* expression domains are very similar and more restricted than *notch3*, specially in Hc and Hd (Fig. 9).

### *notch1a*, *1b* and *notch3* expression in the proliferative areas and radial glia of the adult optic tectum

It has been previously described that, in the optic tectum (TeO) of adult teleost fish, active proliferating cells are located in the dorsomedial area of the periventricular gray zone (PGZ) [8], [17], [19]. These cells give rise to both neurons and glia that are later integrated into the mature tectal layers. This progenitor zone does not display typical radial glia properties as observed in the dorsal telencephalon [17], [19]. Instead, radial glia cells are located in the deep layer of PGZ (dPGZ), lining the ventricle. Another proliferation zone in the mesencephalon is the posterior mesencephalic lamina (PML), that seals the tectal ventricle and extends until the cerebellum [8], [52]. In this study, we refer to the active proliferation areas as the mitotic region of the dorsomedial area of PGZ (mPGZ) and the posterior mesencephalic lamina (PML).

**Figure 10:**
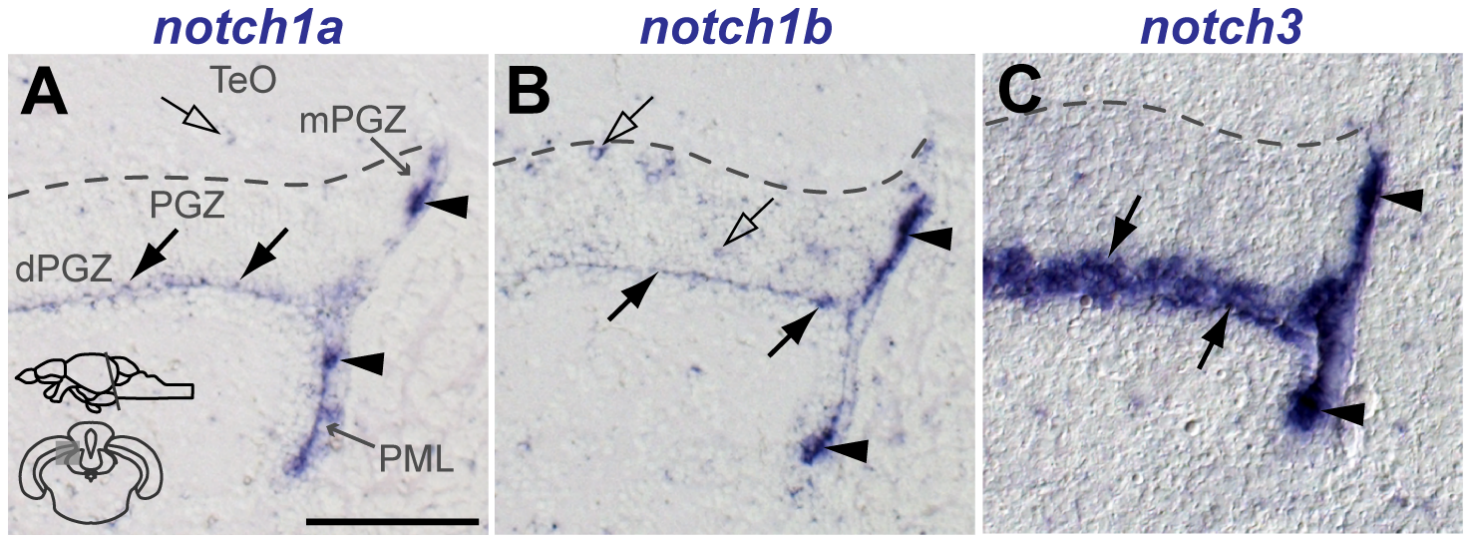
Notch receptor expression in the adult zebrafish optic tectum. Cross-sections at the indicated level through the mesencephalon; optic tectum area shown in the micrographs is indicated in the cross section schematic in A. Brighfield images show the expression (by ISH) of **A**, *notch1a*, **B**, *notch1b* and **C**, *notch3* along the PML and mPGZ (filled arrowheads) and in the dPGZ (filled arrows). A few *notch1a* 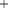 and *notch1b
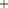
* cells are also found in more superficial layers of the PGZ and TeO (unfilled arrows). Abbreviations: PGZ, periventricular gray zone of the optic tectum; dPGZ, deep layer of the PGZ; mPGZ, mitotic region of the PGZ; PML, posterior mesencephalic lamina; TeO, optic tectum. Scale bars  = 100 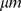 in A (applies to all).

**Figure 11:**
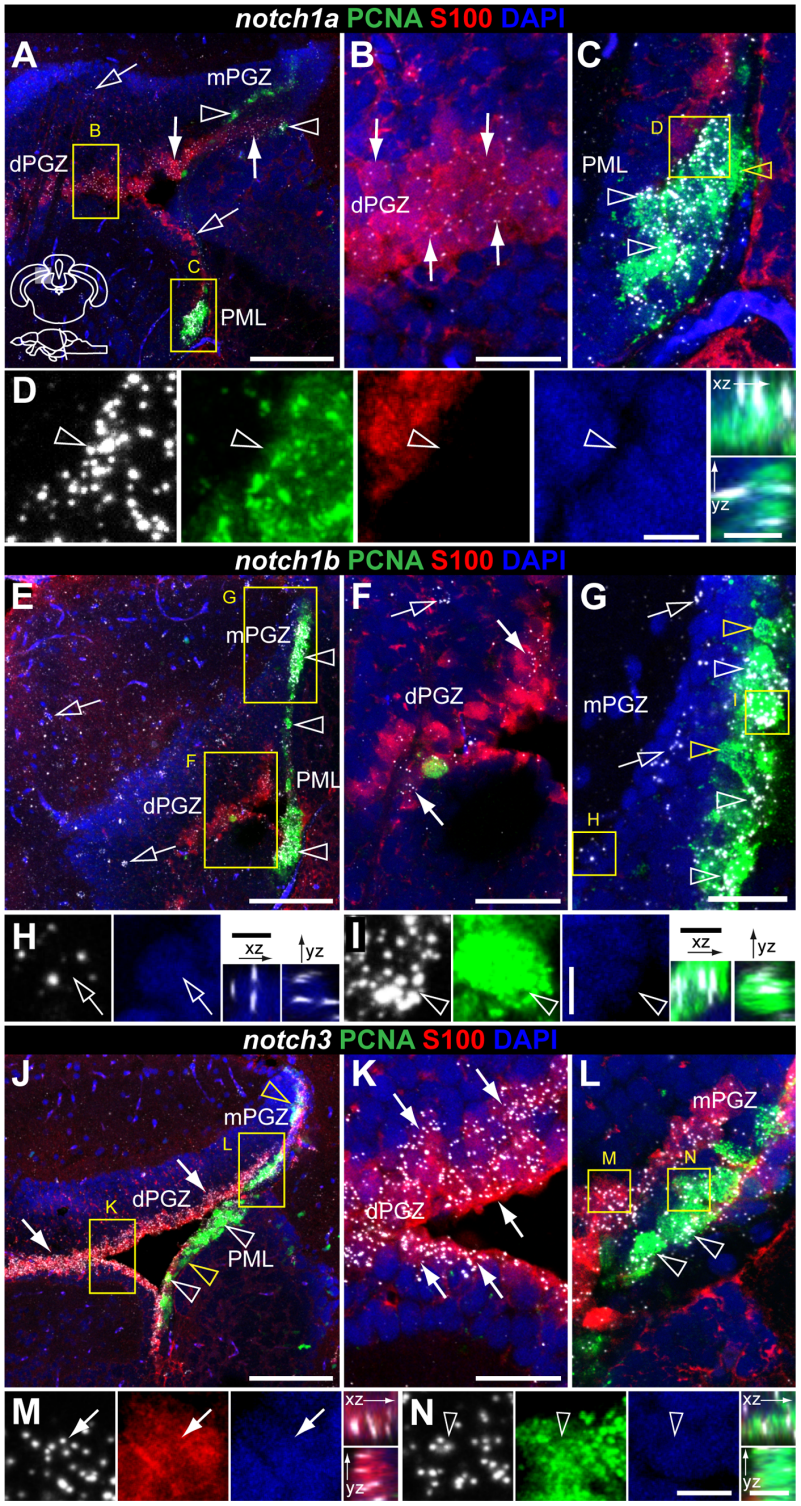
Notch receptor expression in radial glia and proliferating cells of the adult zebrafish optic tectum. Cross-sections at the indicated level through the mesencephalon; optic tectum area shown in the micrographs is indicated in the cross section schematic in A. Confocal images showing localization of the glial marker S100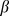 (red) and the proliferation marker PCNA (green), with **A–D**, *notch1a*, **E–I**, *notch1b* and **J–N**,*notch3* by FISH (white). **A**, *notch1a*, **E**, *notch1b* and **J**, *notch3* are present in PCNA 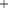 cells of the mPGZ and PML (unfilled white arrowheads) and in S100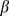 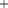 glial cells of the dPGZ; cells in the superficial layer of the PGZ expressing *notch1a* and *notch1b* are also found (unfilled arrows), with relative more abundance for the *notch1b* receptor in this layer; **B, C**, **F,G** and **K,L** are higher magnifications of the respective framed areas in A, E and J, showing the overlap of *notch* expression with S100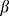 (filled arrows) or PCNA (unfilled white arrowheads); unfilled yellow arrowheads in C, G and J indicate a few Notch receptor 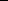 /PCNA 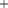 cells; **D**, **H**, **I**, **M**,**N**, close ups of Notch receptor expressing cells in the above mentioned areas and orthogonal views of the indicated cells. Abbreviations: PGZ, periventricular gray zone of the optic tectum; dPGZ, deep layer of the PGZ; mPGZ, mitotic region of the PGZ; PML, posterior mesencephalic lamina. Scale bars  = 100 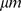 in A, E and J; 20 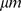 in B (applies to C), in F, G and K (applies to L); 5 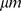 in D, I (applies to H) and N (applies to M).

**Figure 12:**
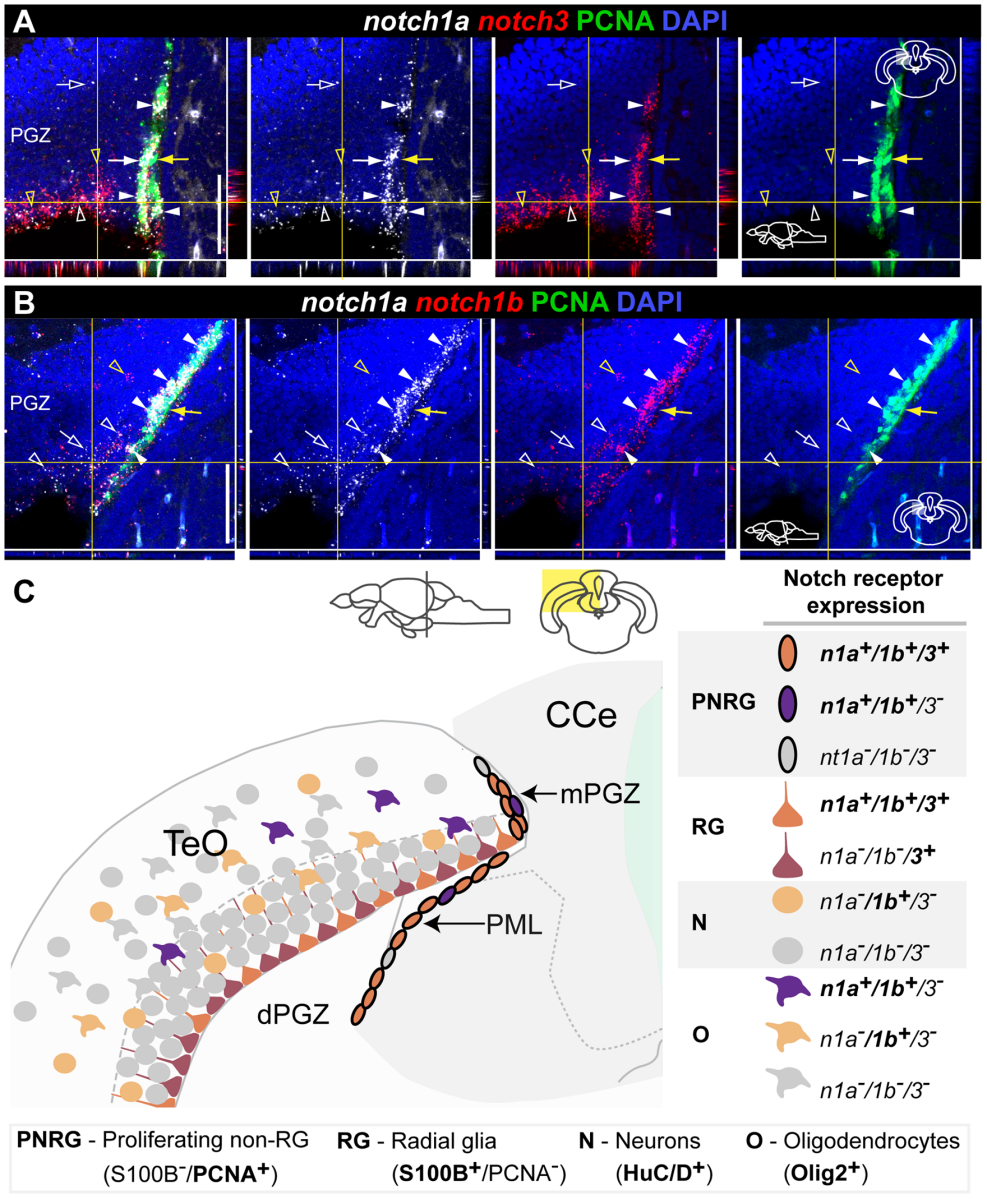
Overlapping and complementary *notch1a/3* expression in the adult zebrafish optic tectum. Confocal images showing localization of Notch receptor pairs by double FISH (white and red) and PCNA 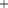 proliferating cells (green). Cross-sections at the indicated level through the mesencephalon; tectal area shown in the micrographs is indicated in the cross section schematics. **A**, *notch1a/3* and **B**, *notch1a/1b* expression domains in the dPGZ and mPGZ layers. Co-expression of these receptors both in PCNA 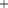 (filled white arrowheads) and PCNA 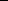 cells (unfilled white arrowheads); *notch3* 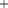 -only cells in A and *notch1b* 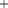 -only cells in B are indicated by unfilled yellow arrowheads; white arrows indicate *notch1a* 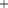 /*notch3* 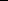 /PCNA 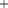 cells in A; a few PCNA 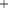 cells are Notch receptor 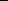 (filled yellow arrows); unfilled white arrows indicate cells positive for *notch1a* alone. **C**, Summary of Notch receptor expression pattern and cellular characteristics in the TeO. Abbreviations: Cce, corpus cerebelli; PGZ, periventricular gray zone of the optic tectum; dPGZ, deep layer of the PGZ; mPGZ, mitotic region of the PGZ; PML, posterior mesencephalic lamina; TeO, optic tectum. Scale bars  = 50 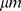.

We observed expression of *notch1a*, *notch1b* and *notch3* in dPGZ, mPGZ and PML (Fig. 10). Both *notch1a* (Fig. 10A) and *notch1b* (Fig. 10B) exhibit a stronger signal in mPGZ and PML than in dPGZ. A few cells in the neuronal layer of the TeO expressed *notch1a* and *notch1b*, the latter having a stronger signal and expression in a higher number of cells (Fig. 10). Similarly to what we observed in the telencephalon, these scattered *notch1a* and *notch1b* cells also express *olig2* (Fig. S6). However, a subpopulation of the *notch1b* 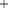 cells in the TeO does not express *olig2* but is positive for the neuronal marker HuC/D (Fig. S6B). *notch3* is strongly and homogeneously expressed in dPGZ, mPGZ and PML (Fig. 10C). Co-localization with the proliferation marker PCNA and the glial marker S100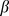 shows that Notch receptors are present in both glia and proliferating cells (Fig. 11). *notch3* is strongly expressed in the majority of S100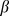 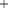 cells (Fig. 11J–M) whereas *notch1a* and *notch1b* expression is scattered in a subset of these cells (Fig. 11B, F). Most proliferating cells in mPGZ and PML express all three Notch receptors (Fig. 11A, E, J). To address if in fact they are co-expressed in the same proliferating cells, or if their expression is complementary, we preformed double FISH. Indeed, only few proliferating cells lack expression of any of the receptors (yellow arrows in Fig. 12A, B). Furthermore, very few PCNA 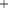 cells expressed either *notch1a* or *notch1b*, but none only *notch3* (white arrows in Fig. 12A, B). In the PGZ S100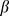 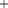 glia, *notch1a* is expressed in a subpopulation of *notch3*-expressing cells (Fig. 12A) while *notch1a/1b* are mostly complementary (Fig. 12B).

In summary, Notch receptors are expressed in the majority of mPGZ and PML cells, *notch3* is expressed in all S100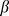 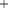 glia of dPGZ, whereas *notch1a* and *notch1b* are in a subset of these cells (Fig. 12C). *notch1a* and *notch1b* are also present in *olig2* 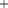 cells of the PGZ and more superficial tectal layers and *notch1b* in some HuC/D 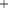 neurons (Fig. 12C). These expression patterns are found throughout the whole rostrocaudal extent of PGZ and PML.

### *notch1a*, *1b* and *notch3* expression in the adult cerebellar niche

**Figure 13:**
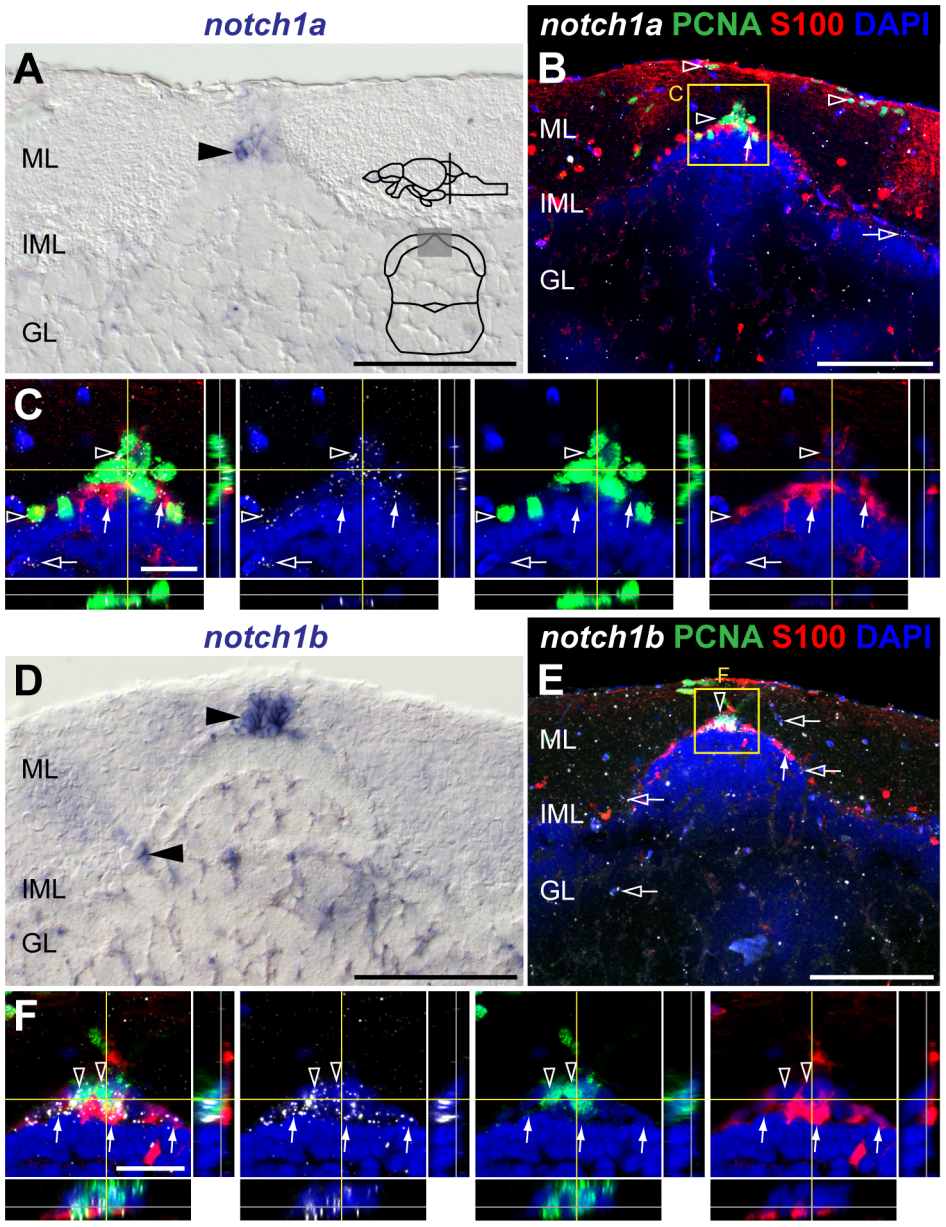
*notch1a* and *notch1b* expression in the adult cerebellar niche. Cross-sections at the indicated level through the mesencephalon; cerebellar area shown in the micrographs is indicated in the cross-section schematic in A. Brightfield images show expression of **A**, *notch1a* and **D**, *notch1b* in the cerebellum (arrowheads). Confocal images showing localization of the glial marker S100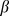 (red) and PCNA (green), with **B–C**, *notch1a* and **E–F**, *notch1b* by FISH (white). **B–C**, Expression of *notch1a* localizes with a large fraction of PCNA 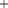 cells in the niche (unfilled arrowheads); weak (white arrow) or no expression of *notch1a* is detected in the Bergmann glia (S100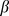 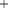). **E–F**, *notch1b* is expressed in a subpopulation of PCNA 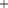 cells (unfilled arrowheads) and in a few S100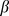 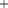 cells (white arrows). A few scattered *notch1a* 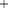 and *notch1b* 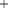 cells in the ML, IML and GL do not localize with the analysed markers (unfilled arrows). Abbreviations: GL, granule cell layer; IML, intermediate layer; ML, molecular layer. Scale bars  = 50 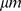 in A, B, D and E; 20 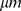 in C and F.

**Figure 14:**
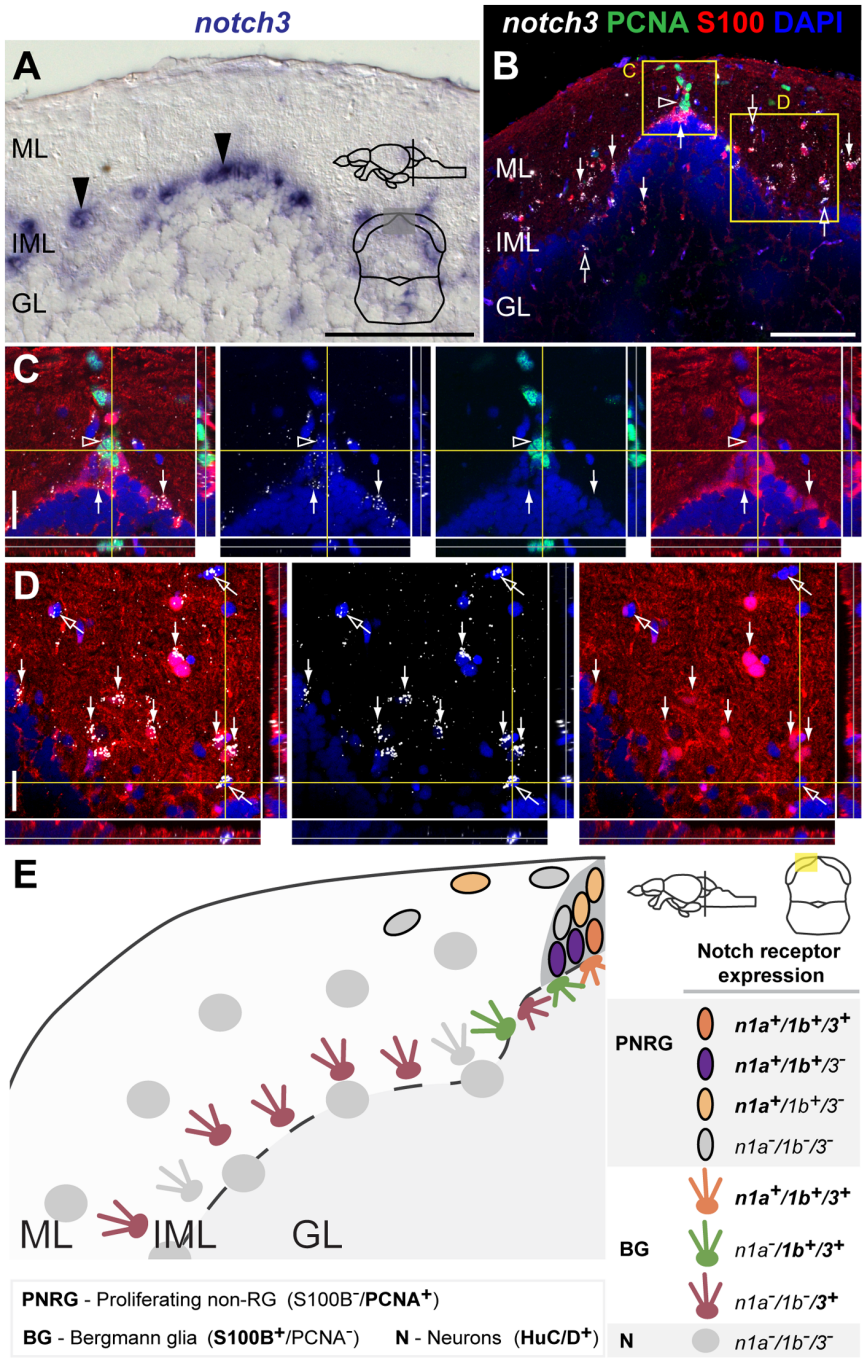
*notch3* expression in the adult cerebellar niche. Cross-sections at the indicated level through the mesencephalon; cerebellar area shown in the micrographs is indicated in the cross-section schematic. **A**, Brightfield image shows *notch3* expressing cells in the cerebellum (black arrowheads). **B–D**, Confocal images showing localization of the glial marker S100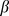 (red) and PCNA (green), with *notch3* by FISH (white). **B–C**, *notch3* is weakly expressed in a small subset of PCNA 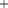 cells in the stem cell niche (unfilled arrowhead). **B–D**, Strong *notch3* expression is detected in S100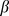 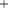 cells indicated by the white arrows; *notch3* expression is also detected in some scattered cells of the ML, IML and GL that do not localize with the analysed markers (unfilled arrows); **E**, Summary of the expression pattern and cellular characteristics of Notch receptor expressing cells in the adult cerebellum. Abbreviations: GL, granule cell layer; IML, intermediate layer; ML, molecular layer. Scale bars  = 50 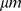 in A and B; 20 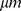 in C and D.

Similarly to the TeO progenitor niche, proliferating cells in the adult cerebellar progenitor niche do not exhibit glial characteristics [15]. Here, progenitors and glia represent two spatially distinct populations. Progenitor cells reside in a cap-like structure of the dorsomedial corpus cerebelli and valvula cerebelli, at the interface between the molecular (ML) and granular cell layer (GL) [15]. We detected *notch1a* and *notch1b* expression in the cap-like structure (Fig. 13A, D). A few *notch1b* 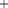 cells are also present in the intermediate molecular layer (IML) (Fig. 13D). *notch3* expressing cells are scattered in the IML, more abundantly than *notch1b*, with a few positive cells in the ML and GL as well (Fig. 14A). We then asked if all three Notch receptors co-localize with PCNA 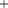 progenitors and S100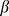 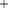 Bergman glia, the latter located in the IML. We observed that *notch1a* and *notch1b* localize with a large portion of PCNA 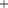 proliferating cells in the cerebellar niche and in a few S100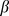 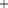 cells, ventrally adjacent to the progenitors (Fig. 13B–F). A few cells positive for *notch1a* or *notch1b* but negative for either PCNA or S100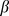 are also detected in the ML, IML and GL (Fig. 13B, E). Co-staining of *notch3* with PCNA and S100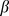 reveals that most proliferating cells lack *notch3* expression while most of the S100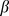 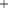 Bergman glia cells express *notch3* (Fig. 14B–D). Also *notch3* expressing cells, negative for either PCNA or S100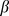 are observed in the ML, IML and GL. We have not observed co-localization of any of the Notch receptors with HuC/D 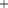 neurons in this region (data not shown).

In summary, *notch1a* is present predominantly in the progenitor niche, whereas *notch1b* is also strongly expressed in a few Bergman glia cells. *notch3* is mostly excluded from the niche but expressed in a high proportion of the Bergman glia (Fig. 14E).

## Discussion

The study of adult neurogenesis and neuronal regeneration in zebrash is fairly recent, thus an understanding of how important developmental pathways are involved in these processes is still missing. The Notch signaling pathway is important both in neural development and in adult neurogenesis, where it has mainly been studied in mammals. In the adult zebrafish brain, the cellular composition within a neurogenic zone and between neurogenic regions is similarly diverse as in mammals. So far, the presence and requirement of Notch signalling in distinct progenitor subtypes or in different steps of the progenitor hierarchy has not been addressed in the adult zebrafish. Thus, a detailed expression analysis of Notch pathway components in this model is important for proper interpretation of further functional studies of this signaling pathway in the context of adult neurogenesis and neuronal regeneration. It has been previously reported that *notch1a/1b/2/3* are expressed in the ventricular zone of the adult zebrafish telencephalon [45], however, no systematic and comparative expression analysis in several neurogenic zones has been done.

**Figure 15:**
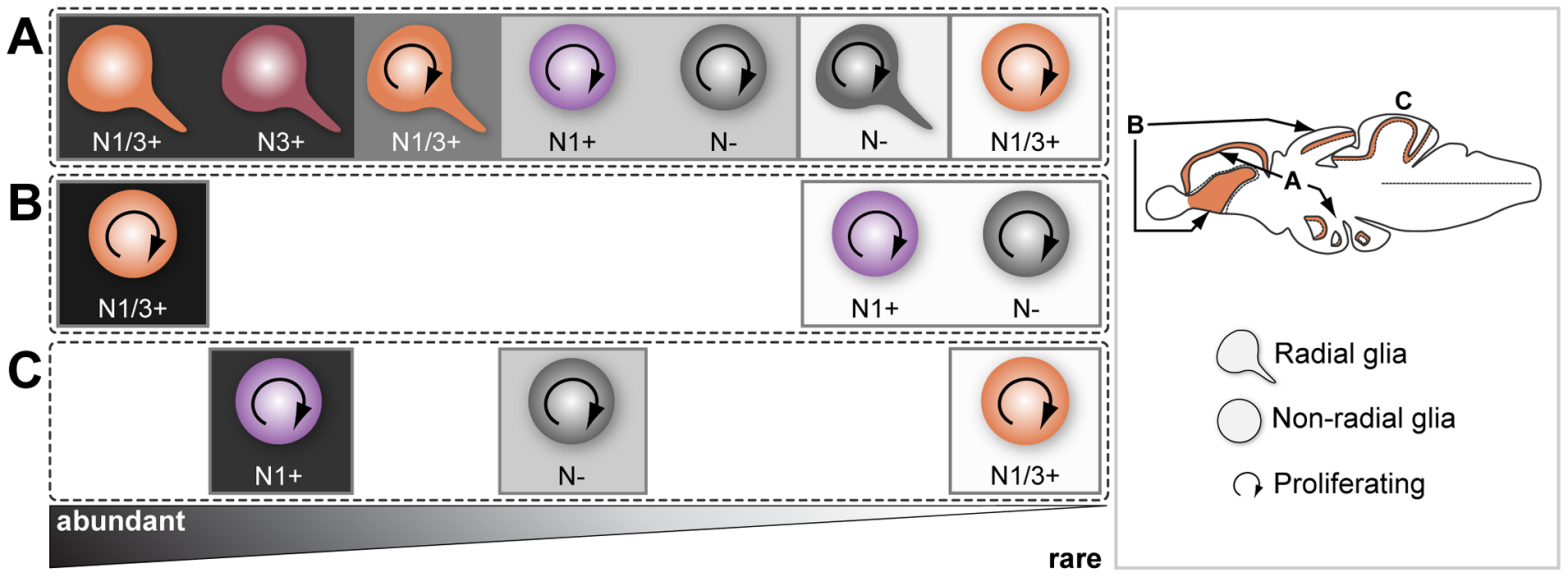
Comparison of Notch receptor expressing cells in the five analysed adult zebrafish neurogenic niches. Notch receptor expression is consistent in a subpopulation of proliferating progenitors or NSCs throughout the adult zebrafish brain. The existing heterogeneity and similarities observed in the combinatorial Notch receptor expression between neurogenic niches might be a reflection of their inherent cellular properties. This is depicted by the presented regional grouping, as indicated on the right side with some regions having similar characteristics. Approximate proportion of the cell populations found is represented by gradient triangle at the bottom, and box around the cells, with decreasing abundance from left (dark) to right (light). Cell populations regarding their Notch receptor expression in the proliferation zone ot the **A**, dorsal telencephalon and hypothalamus **B**, ventral telencephalon and optic tectum and **C** cerebellum. N – Notch receptor; N1 – *notch1a/1b*; N3 – *notch3*.

Here we described in detail the expression pattern of three Notch receptors in five neurogenic regions of the adult zebrash brain, and characterize Notch receptor expressing cells with respect to their proliferative status and glial-type precursor cell identity. Our analysis suggests that the adult zebrafish neurogenic niches have common properties, but also display local and regional heterogeneity regarding Notch receptor expression (Fig. 15). This heterogeneous distribution of Notch receptors could be related to a differential requirement for Notch signaling either in different progenitor types or at different stages/states of progenitor activity.

### Notch signaling in regions of active neurogenesis

In rodents, Notch receptors are expressed in germinal zones where active neurogenesis occurs both in the embryo [40]–[42], [53] and the adult [42], [43], with different receptors showing overlapping and complementary expression patterns [42], [53]. Similarly, we found an extensive overlap in receptor expression patterns in the neurogenic zones of the adult zebrafish brain. In the dorsal zebrafish telencephalon and hypothalamus, proliferating cells negative for the marker S100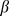 are rarely expressing *notch3* and only half of them are *notch1a/1b*-positive (Fig. 15A). Moreover, in the ventral telencephalon and the mPGZ of the optic tectum, where progenitors have been described to have a neuroepithelial morphology [17], [18], *notch1a*, *notch1b* and *notch3* are present in the majority of proliferating cells and are mostly co-expressed (Fig. 15B). However in the cerebellum, where the neural stem cells also possess neuroepethelial properties [15], *notch3* expression is very weak and observed in very few cells in contrast to *notch1a* and *notch1b* that are expressed in many proliferating cells (Fig. 15C).

Our expression pattern analysis is consistent with what had been observed in zebrafish larval brains, where *notch1a* expression was found to be located ventricularly, together with proliferative cells [54]. In another teleost species, *Carassius auratus* (goldfish), a *notch3* homologue has been identified, and the expression of this receptor was also found in ventricularly located cells of the adult brain [55]. In contrast to what has been described by Chapouton *et al.* [45] we not only observed that *notch3* is expressed in most of the radial glia population of the dorsal telencephalon and hypothalamus neurogenic niches, but also that it is associated, together with *notch1a* and *notch1b*, with proliferating cells throughout the adult brain. A possible explanation for these differences might be that we have preformed the *in situ* hybridization on 12 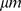 thin sections instead of whole brains [45] which allows for better probe penetration and less probe trapping in ventricular areas. Moreover, we were unable to detect *notch2* expression in the adult brain, opposed to what has been previously reported [45].

In the mammalian brain several studies support the idea that NSCs present high Notch activity, essential for their maintenance and proliferation [26], [27], [56]–[59], whereas neuronal committed progenitors or differentiating neurons display reduced or no Notch activity [26], [59], [60]. Nevertheless, effects on proliferation seem to be dichotomous, tissue- or cell dependent, dose dependent, and receptor dependent [61]–[67]. It is as yet not clear if progenitors in the adult zebrafish brain have distinct developmental origins and which might constitute different progenitor pools, or if there is a common progenitor from which all cell types are derived. It will therefore be interesting to analyze the modification of Notch signaling activity in the adult zebrafish brain on different cell types and diverse differentiation processes. Currently this aspect is difficult to study due to the lack of specific NSC markers and tools to reliably monitor Notch signaling activity in different cell types or at distinct steps of differentiation.

### Notch in different progenitors of the adult zebrafish CNS

In the dorsal telencephalon we observed that approximately 20 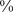 of the radial glia cells are proliferating, in homeostatic conditions. In the adult zebrafish telencephalon at least part of the radial glia population shows high Notch activity [18], [45]. These cells are neurogenic progenitors and increase their proliferative activity upon stab-lesioning to the dorsal telencephalon [68]. In the adult rodent brain, upon injury or stroke, Notch expression in reactive astrocytes increases [34] whereas it decreases in ependymal cells [69]. In ependymal cells, high Notch activity prevents them to re-enter cell cycle in response to injury, limiting their neurogenic potential [69]. In the adult zebrafish, the role of Notch signaling in progenitor proliferation during constitutive neurogenesis [45], [70], [71] and after injury [71], [72] is still unclear, as it might depend on the cellular context.

Here we show that *notch1a*, *notch1b* and *notch3* are expressed in proliferating glial cells, which form the majority of the constitutively proliferating neural progenitor population (Fig. 15A). However, in the glial marker-negative proliferating cells *notch1a* and *notch1b* are found in roughly half of this population, whereas *notch3* expression is rare (Fig. 15A). As the majority of label retaining cells (slow cycling) in the telencephalon are glial marker negative [18], we hypothesise that *notch1a/1b* but not *notch3* might be expressed in a fraction of the slow cycling population (putative NSCs). Considering that the cellular composition of the ventricular zone cells is very diverse, different cell types could be more or less responsive to variations in Notch signaling activity, and the outcome of such variations may be very different depending on the cell type/state and neurogenic niche. It could also well be that combinatorial signaling mediated by the activation of different Notch receptors triggers distinct responses in different cells or at different stages.

### Notch and glial identity

The role of Notch signaling in glial cell fate determination seems to be time and context dependent [27], [33], [73]. During retina development in zebrafish, overexpression of NICD promotes the gliogenic fate at the expense of neurogenesis [74]. In mouse cerebellar development, Notch receptors are expressed in Bergman glia and are important for normal development and maturation of this brain region [75]. This is consistent with our observations that *notch3* is strongly expressed in the majority of S100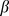 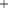 cells, throughout the adult zebrafish brain, with *notch1a* and *notch1b* expressed in a subset of *notch3* 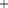/S100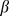 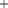 cells. Based on our expression data, we hypothesise that *notch3* might have a more prominent role in the glial lineage, whereas *notch1a/1b* expression might be related to their progenitor character (Fig. 15A). In mammals, both receptor redundancy and receptor sub-functionalization have been proposed [40], [65], [76], however this remains controversial. In zebrafish also little is known in this regard thus functional studies are needed to test whether Notch receptors have a redundant role in adult neurogenesis or not. Based on the diversity of cells and combination of Notch receptors expression found we can speculate that, in the adult zebrafish neurogenic niches, a certain “notch code” might exist resulting in different outcomes for cell fate, stem cell maintenance, cell survival and proliferative capacity.

### Notch receptors and the cell cycle

Progenitors in the ventral part of the ventral telencephalon (Vv) of the adult zebrafish brain show a pseudostratified neuroepithelial-like morphology and interkinetic nuclear migratio [18]. In this region, we observed that Notch receptor expression is stronger in cells closer to the ventricle (apically), where mitosis occurs and decreases away from the ventricle, similar to chick and zebrafish embryonic neuroepithelium [77], [78]. Disruption of the interkinetic nuclear movement resulted in more cells in S-phase and lead to increased neurogenesis, possibly due to a reduced exposure to Notch signaling [77], [79]. In contrast, during mouse brain development, Notch signaling is activated in ventricular zone neuroepithelial cells undergoing S-phase, but not in M-phase cells at the lumenal surface of the ventricular zone [59].

In chick spinal cord, a study reports that Notch activity increases at a higher frequency prior to mitosis [80]. However, there are also cells that activate Notch signaling after mitosis, while they move basally, either in only one sibling or in both, suggesting that Notch activation at different cell cycle phases might be related with the cell fate of daughter cells [80]. Conceivably, the situation in Vv may be similar to the chick neural tube, where divisions resulting in two daughter cells with similar Notch activity stay progenitors while siblings with an asymmetric Notch activity adopt different fates (Fig. 15B). However, additional studies looking at Notch signaling activity in the ventricular zone of Vv will be necessary.

### Notch receptor expression outside proliferation zones

Notch signaling is not only active in germinal zones but also in non-proliferative areas. Similarly to the adult mouse cerebellum [43], *notch3*, and partially *notch1b*, are expressed in Bergmann glia in the adult zebrafish. Additionally, *Notch1* receptor is expressed in mature neurons in the rodent cortex [35], [42], [81] where it is necessary for proper neocortical neuronal migration [81]. Moreover, it has been shown that Notch signaling regulates dendrite growth [82]–[85] and morphology [35], [81]. Interestingly, during zebrafish spinal cord neurogenesis, *notch1a* is expressed both in neural precursors and in differentiating neurons whereas *notch1b* and *notch3* are found only in neural precursors [86]. In contrast, in the adult zebrafish brain we observe expression of all three receptors in putative neuronal precursors but only *notch1b* in a HuC/D 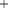 neuronal population. However, we did not detect Notch receptor expression in new born neurons adjacent to germinal zones. The *notch1b* expression that we observed in neurons could indicate that also in the adult zebrafish brain Notch signaling might serve later stages of neuronal differentiation and/or neuronal activity.

Oligodendrocytes are another cell type produced in neurogenic regions of the adult zebrafish brain [17], [18], [68], [87]. *olig2* is a marker for both oligodendrocyte precursor cells (OPCs) and, in combination with other markers, for more mature oligodendrocytes [18], [87]–[90]. Interestingly, we also found Notch receptor expression in a subpopulation of *olig2* 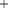 cells in the telencephalon parenchyme and optic tectum. We also detected Notch receptor expression in *olig2* 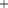 proliferating cells in the ventricular zone (not shown), but the parenchyma subpopulation of Notch receptor 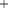 /*olig2* 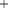 cells were not proliferating. It has been shown that Notch signaling, together with *olig2*, promotes the specification of OPCs [38] but inhibits their differentiation [36], [37]. Assuming that Notch signaling has a similar role in adult oligodendrogenesis, this parenchymal Notch receptor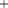/*olig2
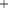
* subpopulation could represent immature oligodendrocytes.

Notch signaling is also involved in the development of the vascular system (see review(s) [91], [92]). Notch1 has been implicated in vascular homoeostasis [91], and in zebrafish, *notch3* expression is associated with the arterial fate during arterial-venous differentiation [93]. In the present study, we observed a subset of endothelial cells in the telencephalon that express *notch1a* and *notch3*. This suggests that also in the adult zebrafish brain, Notch signaling might be important for vessel homeostasis or for the maintenance of arterial phenotype in some cells.

## Conclusion

Our results showed that Notch receptor expression domains overlap to a great extent in the neurogenic areas, but also that regional and cellular heterogeneity in the expression of different receptors exists in the adult zebrafish brain. The differences observed between receptor expression in certain niches could indicate that they are involved in distinct cellular processes. Therefore, we hypothesise that: i) predominantly *notch3* might be important for the glial properties of progenitors as well as other glial cells; ii) *notch1a/1b* might modulate progenitors proliferative status in a level-dependent manner [67]; iii) combinatorial Notch receptor expression could be necessary to achieve a certain basal level of Notch activity, required for NSC maintenance; iv) also in the adult brain, Notch signalling might be involved in multiple processes including neuronal maturation, oligodendrogenesis and blood vessel homeostasis. All these hypothesis remain to be tested functionally in the context of adult neurogenesis in the zebrafish brain. Only cell type specific modulation of the pathway will allow to unravel the role of Notch signalling in these processes. Moreover, labelling and lineage tracing of different progenitor cells are needed to achieve a better understanding of the ontogeny, properties and persistence of neurogenic niches in adulthood.

## Materials and Methods

### Ethics statement

All procedures were in accordance with the live animal handling and research regulations of the University and State of Saxony, Germany, review boards, the Regierungspräsidium Dresden (permit AZ 24D-9168.11-1/2008–1 and −4). This institutional review board specifically approved this study.

### Animals and tissue preparation

Fish were kept under standard conditions as previously described [94], [95]. Wildtype experimental animals were adult fish from the *gol-b1* line in the AB genetic background [96]. Adult fish were 6 months old and both sexes were used in this study. Transgenic animals Tg(*fli*1:GFP) [48] were 1.5 years old.

Whole heads with the brain exposed were fixed in 4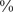 PFA/0.1 M phosphate buffer pH 7.4, at 4°C overnight. After fixation they were washed twice with 0.1 M phosphate buffer, pH 7.4 and transferred into 20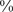 sucrose/20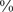 EDTA in 0.1 M phosphate buffer, pH 7.4, at 4°C overnight for decalcification and cryoprotection. Heads were embedded and frozen in 7.5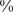 gelatine/20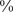 sucrose. For chromogenic *in situ* hybridization experiments, brains were cryosectioned in 14 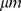 sections. For fluorescent *in situ* hybridization (FISH) stainings, brains were cryosectioned in 12 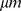 sections. Sections were collected on superfrost slides. After sectioning the slides were stored at −20°C.

### *In situ* hybridization and FISH

Riboprobe preparation was done as previously described [97], using the NTP labelling mixture (Roche). In this study we used the following probes: *notch1a* [46] (2.7 kb, nucleic position 3027–5812 of NM 131441.1), *notch1b* [47] (1.3 kb, nucleic acid position 4738–6080 of NM_131302.2), *notch2* [47] (3.2 kb of NM 001115094.1), *notch3* [47] (1.3 kb, nucleic acid position 4724–5985 of NM 131549.2) and *olig2* [98]. The *in situ* hybridization was done as previously described [97] with some modifications. Hybridization was done at 60°C followed by stringent washes at 62°C. Sections were incubated with either anti-digoxigenin-AP (#11093274910, Roche) or anti-fluorescein-AP (#11426338910, Roche) Fab fragment diluted 1∶4000 and subsequently stained with the substrate NBT/BCIP (Roche). In the case of FISH, a similar protocol was followed but with some modifications. Briefly, after thawing the sections, they were rehydrated in PBS, and then incubated in 0.3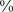 H
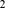
O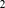 for 30 minutes. After quenching, the tissue was post-fixed in 4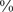 PFA for 20 minutes. Prior to hybridization, sections were washed in PBS and then in PBSTx. For the blocking step, sections were incubated for 1 hour at RT in 1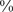 of blocking reagent (Perkin-Elmer) in MABT. After blocking, sections were incubated overnight at 4°C in anti-digoxigenin-POD, or anti-fluorescein-POD Fab fragment (Roche), diluted 1∶500. For detection we used the TSA Plus Cy3/Cy5 kit (NEL744001KT/ NEL745001KT, Perkin-Elmer). The FISH shows the same expression pattern as in the chromogenic *in situ* reaction, with better sensitivity for detecting cells expressing lower levels of *notch* transcripts. All *in situ* hybridizations were done on at least five individuals. All probes were tested on embryos to confirm probe specificity. We have also perfomed negative controls: 1) with probe and without anti-DIG antibody, and 2) without probe and with anti-DIG antibody to assess for unspecific signal (antibody or tyramid trapping). In these tests no signal was detected under the fluorescence microscope.

### Immunohistochemistry

Immunostainings for S100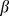 (polyclonal rabbit, Dako Cyto., 1∶500), PCNA (monoclonal mouse, molecular probes, 1∶1000) and HuC/D (monoclonal mouse, molecular probes, 1∶200) were done after completion of the FISH protocol. For PCNA and HuC/D stainings we retrieved the antigen in 50 mM Tris Buffer, pH 8, for 8 minutes at 95°C and 15 minutes at RT, prior to primary antibody incubation. As previously d escribed [8], after primary antibody incubation we washed the sections 3 times in PBSTx, and incubated in species-specific Alexa 488-, 555-, and 633-conjugated secondary antibodies (Invitrogen). All immunostainings were done on at least three individuals.

### Image acquisition and processing

Brightfield and DIC images were taken with the Zeiss Axio Imager microscope (objectives Zeiss Plan-Apochromat 10×/0.45 Dry, Zeiss Plan-Apochromat 20×/0.8 Dry and Zeiss Plan-Apochromat 40×/0.95 Water). Confocal images were taken on a Leica TCS-SP5, using the Leica HCX PL APO 40×/0.75 Dry and Leica HCX PL APO 63×/1.2 Water objectives. Image acquisition for the quantifications was done using the Leica HCX PL APO 40×/0.75 Dry objective. To minimize cross talk between the channels, sequential image acquisition was performed. Images were processed using Fiji (http://fiji.sc), CombineZP v7.0 (http://www.hadleyweb.pwp.blueyonder.co.uk/CZP/News.htm) and Adobe Photoshop CS4. Figures were assembled using Adobe Illustrator CS4. Confocal images shown are single planes or maximum projections of up to 3 planes (maximum 5 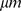 thick, corresponding to only 1 cell layer). Co-localization analysis of the different markers was done in the Z-stack using the orthogonal view tool in Fiji. We considered a positive cell for Notch receptor expression when at least 2 dots of tyramid precipitate where observed in more than one plane of the cell.

### Cell counting and statistical analysis

The quantifications of co-localization between the FISH, S100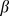 and PCNA markers were preformed in a total of 9 individuals, using 6 sections (12 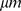) per fish, representative of main telencephalon parts along the rostrocaudal axis. In these quantifications we subdivided the dorsal telencephalon into dorsal-medial and dorsal-lateral areas. Co-localization of the markers was analysed through each z-stack using the cell counter plugin and the orthogonal view tool in Fiji, with the aid of DAPI nuclear counterstaining and S100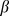, glial marker that labels cell cytoplasm. Statistical significance analysis was done in the statistical computing software R (http://www.r-project.org/) using ANOVA followed by a post-hoc Tukey's HSD test.

### Fluorescence measurements and statistical analysis

To analyse Notch receptor expression levels in proliferating cells of the ventral telencephalon we measured the mean grey value (MGV) of the FISH signal for each receptor. We selected the regions of interest based on the position of the PCNA 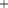 nucleus (apical or basal). A nucleus was considered apical if positioned immediately adjacent to the ventricular surface; as basal if the nucleus was between 2
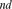
–4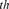 rows of nuclei away from the ventricle. We measured the MGV in selected apical- or basal-area of 2–6 tissue sections of the rostro-ventral telencephalon. To each measurement we subtracted the background signal within the tissue. After background subtraction, MGVs were normalized against the maximum MGV of each experiment. In total, 28 apical-area and 28 basal-area MGVs were obtained for *notch1a* (n = 4), 24 for *notch1b* (n = 4) and 26 for *notch3* (n = 5). For data visualization and median comparison we used notched boxplots in R and modified them with Adobe Illustrator CS4. To confirm statistically significant differences in expression of each gene between apical and basal or between genes in the same nuclei position we applied the Wilcoxon rank-sum test or ANOVA followed by a post-hoc Tukey's HSD test, respectively. Fluorescence profile analysis was done using Fiji Plot Profile. Fluorescence values for each gene expression were normalized for background signal in the tissue and their corresponding maximum. Plots were done in Microsoft Ofice Excel 2007 and modified in Adobe Illustrator CS4.

## Supporting Information

Figure S1
**Notch receptor expression in telencephalic oligodendrocytes.** Confocal images of double FISH showing the localization of Notch receptor (white) and *olig2* (green) in the dorsal telencephalon parenchyme. **A–C**, *notch1a*, *notch1b* and *notch3* are expressed in a subpopulation of parenchymal olig2 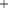 cells (white arrows). Yellow arrows indicate Notch receptor 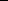 /*olig2* 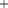 cells; unfilled white arrows in B indicate *notch1b* 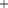 /*olig2* 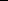 cells. Scale bar  = 50 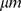 in A (applies to all).(TIF)

Figure S2
**Notch receptor expression in** ***fli*** **1:gfp endothelial cells in the telencephalic parenchyme.** Confocal images showing localization of *notch1a* and *notch3* and gfp 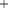 endothelial cells in the dorsal telencephalon parenchyme. **A–B** *notch1a* and *notch3* expression in a few endothelial cells (white arrows); unfilled arrow in B indicates a *notch3* 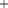 /gfp 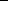 cell adjacent to the blood vessel. Scale bar  = 20 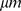 in A (applies to B).(TIF)

Figure S3
**Apical to basal gradient of Notch receptor expression in the ventral telencephalic niche.** Fluorescence profile measurements of Notch receptor expression in the two indicated areas (dark grey rectangle in schematics) of the Vv proliferation zone. Stronger fluorescence signals are detected in proliferating cells (PCNA 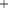) with a more apical nucleus. **A**, Where the proliferation zone is thinner (only 2 PCNA 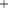 nuclei), all three receptors show a steep profile with comparatively low levels of expression in the more basal proliferating cell. **B**, Where the proliferation zone is thicker (3 PCNA 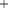 nuclei), *notch3* is mostly expressed in the cell closer to the ventricle whereas *notch1a* and *notch1b* show strong expression levels until 2 PCNA 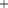 nuclei away from the ventricle. These measurements were done in single stacks corresponding to the images shown in Fig. 5A–C.(TIF)

Figure S4
***notch3*** **expression in glia and proliferating cells of the adult zebrafish hypothalamus.** Confocal images showing localization of *notch3* mRNA by FISH (white), radial glia labelled with S100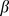 (red), and PCNA 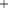 proliferating cells (green). Cross-sections at the indicated levels through the diencephalon; hypothalamic area shown in the micrographs is indicated in the cross-section schematics. **A, B**, *notch3* is expressed in most PCNA 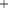/100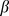 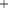 cells (filled white arrowheads) and in PCNA 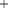/S100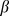 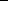 cells (unfilled white arrowheads) of the Hv; unfilled yellow arrowheads indicate *notch3* 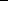 /PCNA 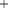/S100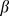 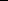 cells. **A–F**, *notch3* localizes with most S100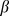 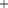 cells of Hd and Hc, PCNA 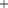 (filled white arrowheads) or PCNA 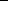 (filled white arrows); filled yellow arrowhead indicates a *notch3* 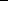 /PCNA 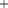/S100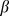 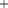 cell. Notice the *notch3* expression in the S100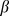 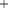 cellular processes in Hc (in E). Asterisk indicates a S100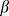 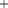 group of cells in Hd that is negative for *notch3*. Abbreviations: Hc, caudal zone of the periventricular hypothalamus; Hd, dorsal zone of the periventricular hypothalamus; Hv, ventral zone of the periventricular hypothalamus: PR, posterior recess of the diencephalic ventricle. Scale bar  = 100 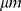 in A and D, 20 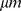 in B, B 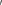, C, C 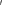, E, F, F 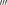.(TIF)

Figure S5
**Overlapping and complementary** ***notch1a/3*** **expression in the adult zebrafish hypothalamus.** Confocal images of double FISH showing the localization of *notch1a* (white), *notch3* (red), and PCNA (green). Cross-sections at the indicated level through the diencephalon; hypothalamic area shown in the micrographs is indicated in the cross-section schematics. **A–C**, *notch1a* is expressed in a subpopulation of *notch3* 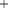 /PCNA 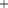 cells in Hv, Hd and Hc (filled white arrowheads); yellow arrows indicate Notch receptor 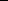 /PCNA 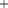 cells; unfilled yellow arrowheads indicate cells expressing *notch3* 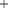 alone. *notch1a* expression partially overlaps with the *notch3* 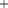 /PCNA 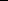 population (unfilled white arrowheads); there are a few *notch1a* 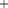 /*notch3* 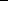 /PCNA 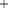 cells in Hv and Hd (filled white arrows). Abbreviations: Hc, caudal zone of the periventricular hypothalamus; Hd, dorsal zone of the periventricular hypothalamus; Hv, ventral zone of the periventricular hypothalamus. Scale bar  = 100 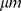 in A (applies to B).(TIF)

Figure S6
***notch1a*** **and** ***notch1b*** **expression in olig2 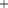 and HuC/D 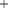 cells of the optic tectum.** Confocal images of double FISH showing the localization of *notch1a/1b* (white), *olig2* (green) and HuC/D (red) in the superficial layer of the optic tectum. Cross-sections at the indicated level through the mesencephalon; tectal area shown in the micrographs is indicated in the cross section schematic in A. **A–B**, *notch1a* and *notch1b* are expressed in a subpopulation of *olig2* 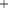 cells (white arrows); yellow arrows indicate Notch receptor 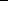 /*olig2* 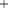 cells. **B**, *notch1b* is also expressed in *olig2* 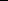 /Hu 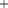 cells (unfilled white arrowheads). Scale bars  = 50 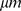.(TIF)

Table S1
**Localization of Notch receptor positive cells with glial and proliferation markers.** Number of cells counted in adult zebrafish Dm and Dl telencephalic areas according to their notch expression and co-label with radial glial (S100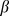) and proliferation (PCNA) markers. nd, not determined; 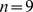.(PDF)

Video S1 **3D animation showing** ***notch1a*** **expression in proliferating and non-proliferating glial cells of the dorsal telencephalon.** This animation corresponds to the z-stack containing the images shown in Fig. 2B.
(AVI)

Video S2 **3D animation showing** ***notch1b*** **expression in proliferating and non-proliferating glial cells of the dorsal telencephalon.** This animation corresponds to the z-stack containing the images shown in Fig. 2E.
(AVI)

Video S3 **3D animation showing** ***notch3*** **expression in proliferating and non-proliferating glial cells of the dorsal telencephalon.** This animation corresponds to the z-stack containing the images shown in Fig. 2H.
(AVI)
