# Supplementary figures and images for: Notch Receptor Expression in Neurogenic Regions of the Adult Zebrafish Brain
Source: PLoS One. 2013 Sep 9;8(9):e73384. doi: 10.1371/journal.pone.0073384 (PMC3767821; doi:10.1371/journal.pone.0073384)

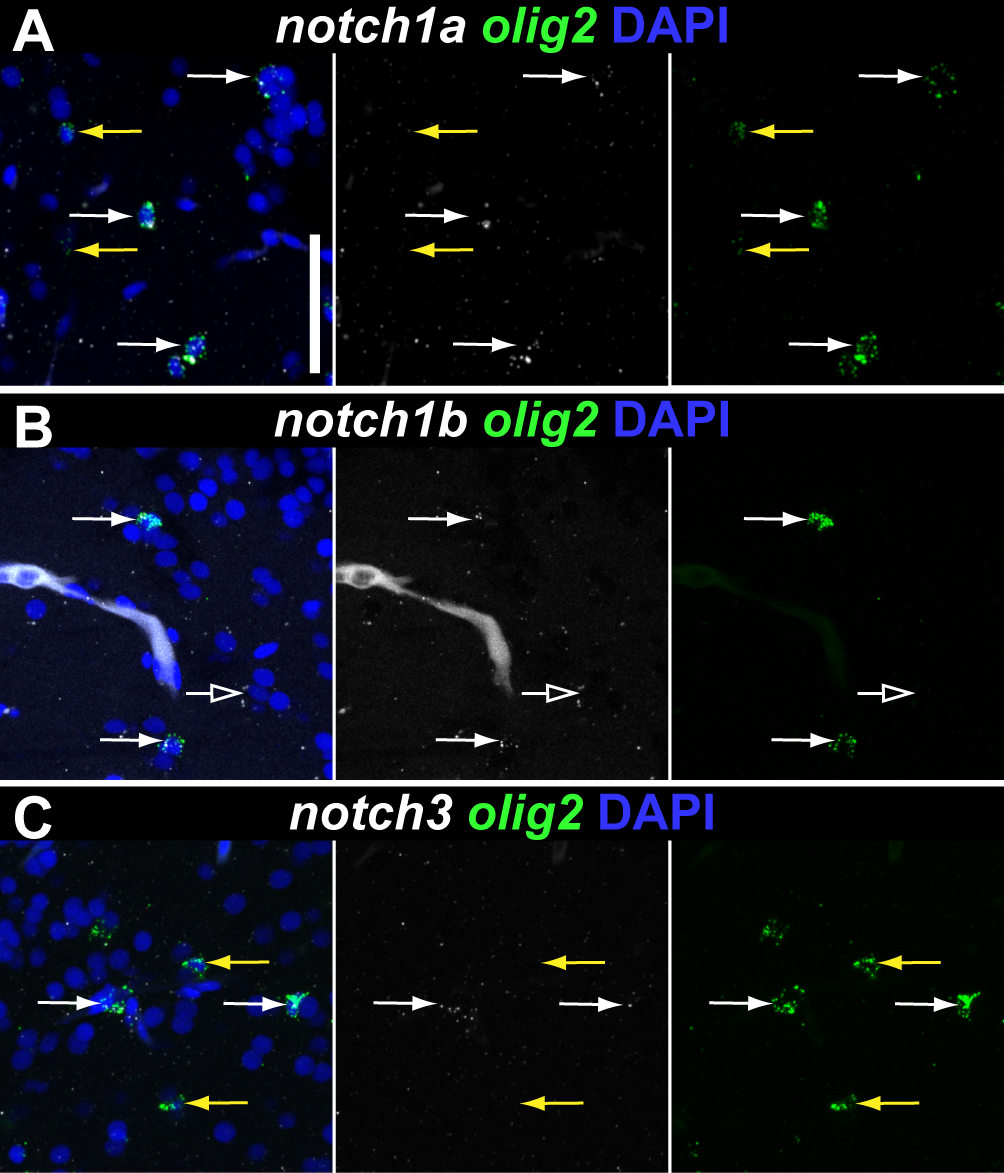

Supplement: Figure S1 — Notch receptor expression in telencephalic oligodendrocytes. Confocal images of double FISH showing the localization of Notch receptor (white) and olig2 (green) in the dorsal telencephalon parenchyme. A–C, notch1a, notch1b and notch3 are expressed in a subpopulation of parenchymal olig2 cells (white arrows). Yellow arrows indicate Notch receptor /olig2 cells; unfilled white arrows in B indicate notch1b /olig2 cells. Scale bar = 50 in A (applies to all). (TIF) [file pone.0073384.s001.tif]

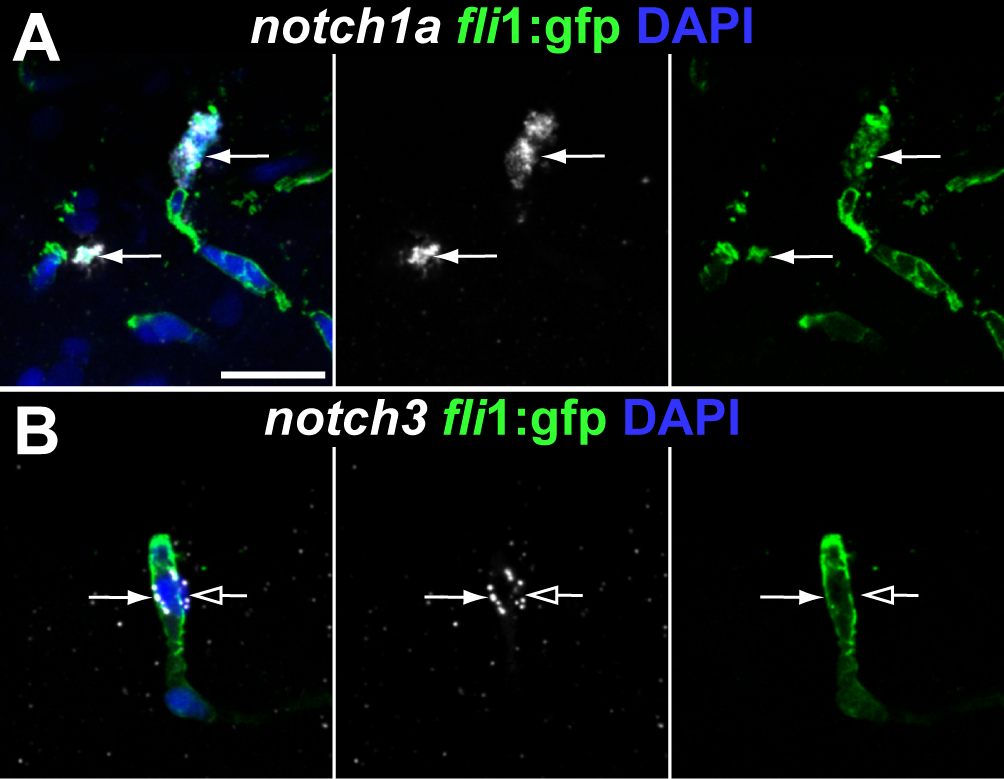

Supplement: Figure S2 — Notch receptor expression in fli 1:gfp endothelial cells in the telencephalic parenchyme. Confocal images showing localization of notch1a and notch3 and gfp endothelial cells in the dorsal telencephalon parenchyme. A–B notch1a and notch3 expression in a few endothelial cells (white arrows); unfilled arrow in B indicates a notch3 /gfp cell adjacent to the blood vessel. Scale bar = 20 in A (applies to B). (TIF) [file pone.0073384.s002.tif]

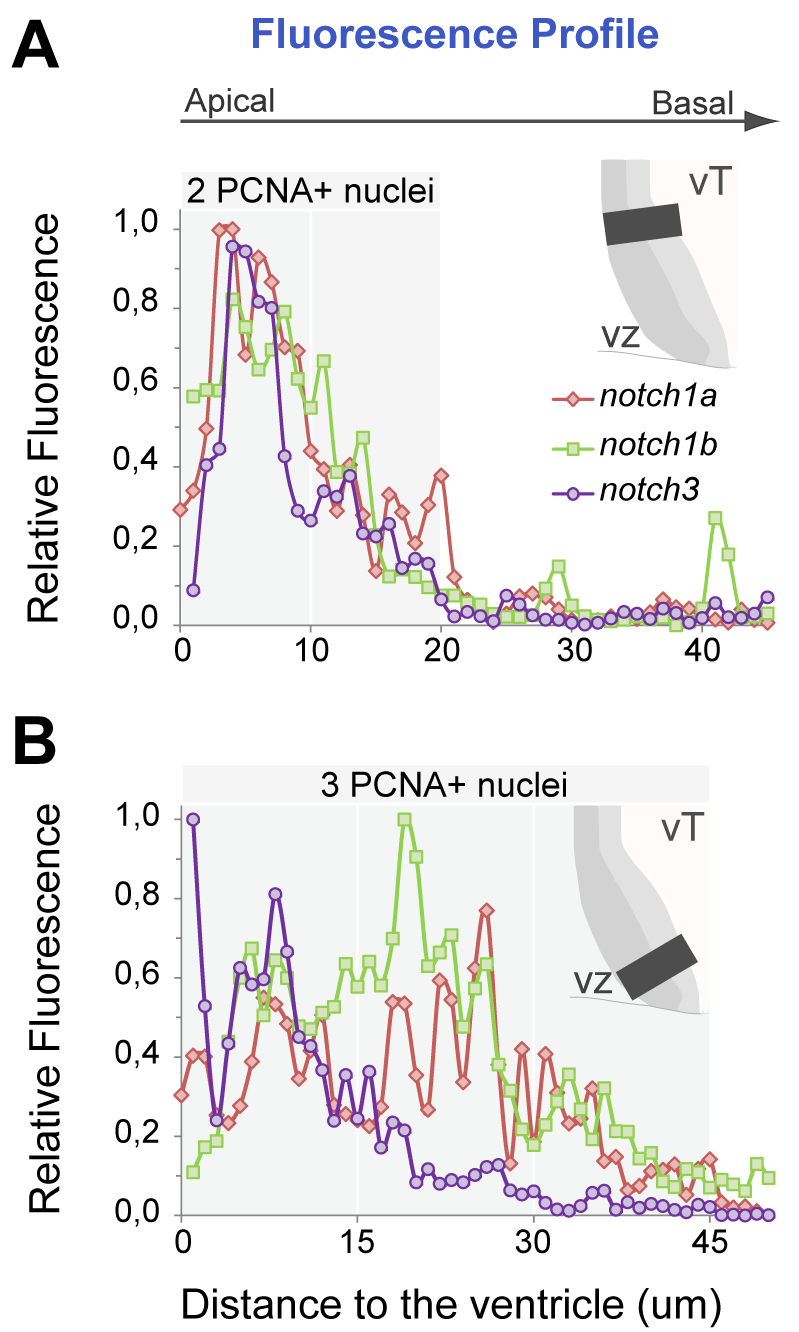

Supplement: Figure S3 — Apical to basal gradient of Notch receptor expression in the ventral telencephalic niche. Fluorescence profile measurements of Notch receptor expression in the two indicated areas (dark grey rectangle in schematics) of the Vv proliferation zone. Stronger fluorescence signals are detected in proliferating cells (PCNA ) with a more apical nucleus. A, Where the proliferation zone is thinner (only 2 PCNA nuclei), all three receptors show a steep profile with comparatively low levels of expression in the more basal proliferating cell. B, Where the proliferation zone is thicker (3 PCNA nuclei), notch3 is mostly expressed in the cell closer to the ventricle whereas notch1a and notch1b show strong expression levels until 2 PCNA nuclei away from the ventricle. These measurements were done in single stacks corresponding to the images shown in Fig. 5A–C. (TIF) [file pone.0073384.s003.tif]

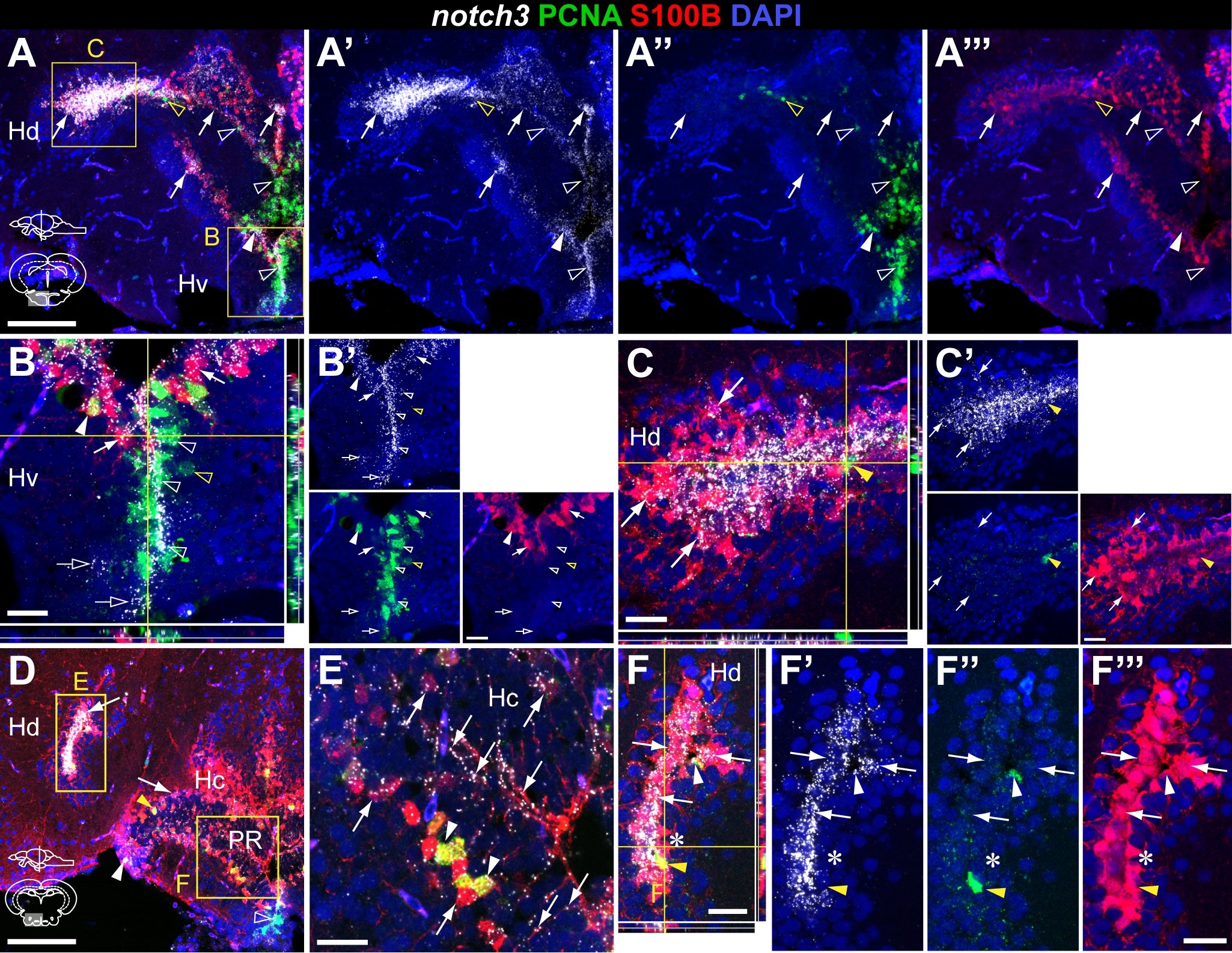

Supplement: Figure S4 — notch3 expression in glia and proliferating cells of the adult zebrafish hypothalamus. Confocal images showing localization of notch3 mRNA by FISH (white), radial glia labelled with S100 (red), and PCNA proliferating cells (green). Cross-sections at the indicated levels through the diencephalon; hypothalamic area shown in the micrographs is indicated in the cross-section schematics. A, B, notch3 is expressed in most PCNA /100 cells (filled white arrowheads) and in PCNA /S100 cells (unfilled white arrowheads) of the Hv; unfilled yellow arrowheads indicate notch3 /PCNA /S100 cells. A–F, notch3 localizes with most S100 cells of Hd and Hc, PCNA (filled white arrowheads) or PCNA (filled white arrows); filled yellow arrowhead indicates a notch3 /PCNA /S100 cell. Notice the notch3 expression in the S100 cellular processes in Hc (in E). Asterisk indicates a S100 group of cells in Hd that is negative for notch3. Abbreviations: Hc, caudal zone of the periventricular hypothalamus; Hd, dorsal zone of the periventricular hypothalamus; Hv, ventral zone of the periventricular hypothalamus: PR, posterior recess of the diencephalic ventricle. Scale bar = 100 in A and D, 20 in B, B , C, C , E, F, F . (TIF) [file pone.0073384.s004.tif]

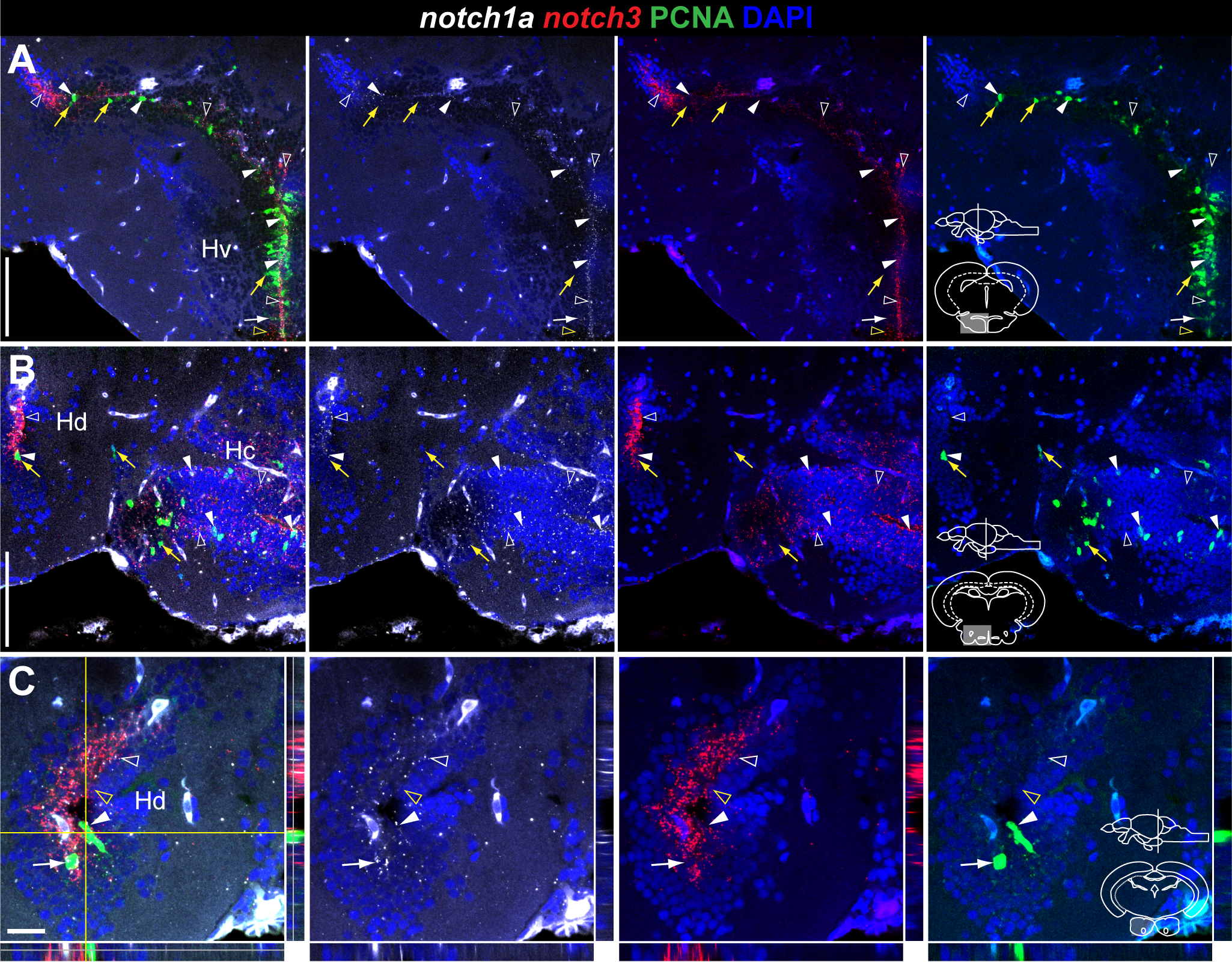

Supplement: Figure S5 — Overlapping and complementary notch1a/3 expression in the adult zebrafish hypothalamus. Confocal images of double FISH showing the localization of notch1a (white), notch3 (red), and PCNA (green). Cross-sections at the indicated level through the diencephalon; hypothalamic area shown in the micrographs is indicated in the cross-section schematics. A–C, notch1a is expressed in a subpopulation of notch3 /PCNA cells in Hv, Hd and Hc (filled white arrowheads); yellow arrows indicate Notch receptor /PCNA cells; unfilled yellow arrowheads indicate cells expressing notch3 alone. notch1a expression partially overlaps with the notch3 /PCNA population (unfilled white arrowheads); there are a few notch1a /notch3 /PCNA cells in Hv and Hd (filled white arrows). Abbreviations: Hc, caudal zone of the periventricular hypothalamus; Hd, dorsal zone of the periventricular hypothalamus; Hv, ventral zone of the periventricular hypothalamus. Scale bar = 100 in A (applies to B). (TIF) [file pone.0073384.s005.tif]

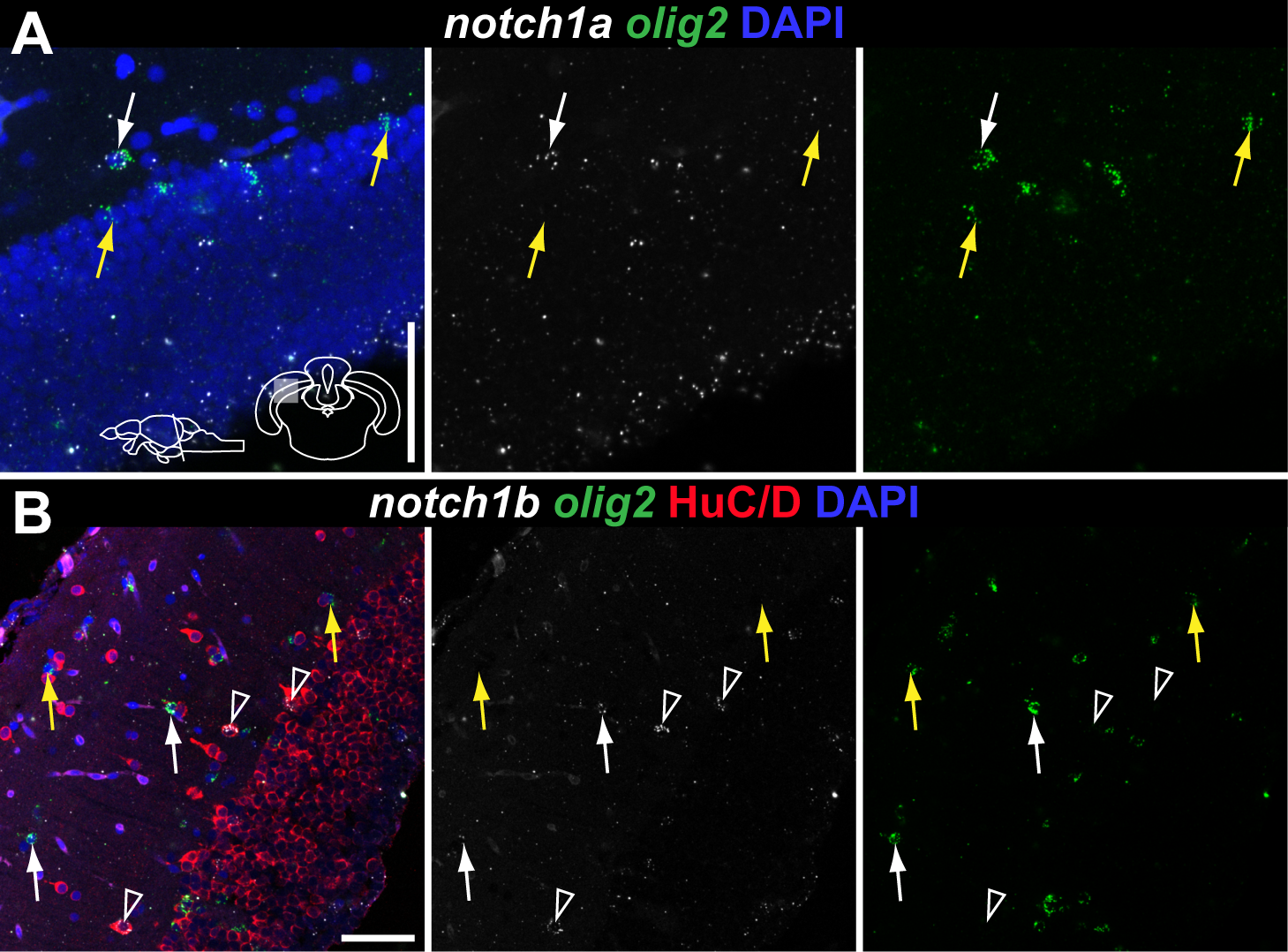

Supplement: Figure S6 — notch1a and notch1b expression in olig2 and HuC/D cells of the optic tectum. Confocal images of double FISH showing the localization of notch1a/1b (white), olig2 (green) and HuC/D (red) in the superficial layer of the optic tectum. Cross-sections at the indicated level through the mesencephalon; tectal area shown in the micrographs is indicated in the cross section schematic in A. A–B, notch1a and notch1b are expressed in a subpopulation of olig2 cells (white arrows); yellow arrows indicate Notch receptor /olig2 cells. B, notch1b is also expressed in olig2 /Hu cells (unfilled white arrowheads). Scale bars = 50 . (TIF) [file pone.0073384.s006.tif]
