# Supplementary material for: Notch Receptor Expression in Neurogenic Regions of the Adult Zebrafish Brain
Source: PLoS One. 2013 Sep 9;8(9):e73384. doi: 10.1371/journal.pone.0073384 (PMC3767821; doi:10.1371/journal.pone.0073384)
Supplement: Table S1 — Localization of Notch receptor positive cells with glial and proliferation markers. Number of cells counted in adult zebrafish Dm and Dl telencephalic areas according to their notch expression and co-label with radial glial (S100) and proliferation (PCNA) markers. nd, not determined; . (PDF) [file pone.0073384.s007.pdf]

	Notch receptor	S100β^+/PCNA$^+$	S100β^+/PCNA$^-$	S100β^-/PCNA$^+$	S100β^-/PCNA$^-$
Dm	*notch1a*$^+$	394	941	72	12
	notch1a$^-$	39	918	46	nd
	notch1b$^+$	346	1024	59	9
	notch1b$^-$	64	838	46	nd
	notch3$^+$	369	1818	26	6
	notch3$^-$	43	60	115	nd
Dl	*notch1a*$^+$	261	938	19	9
	notch1a$^-$	41	906	30	nd
	notch1b$^+$	259	999	45	18
	notch1b$^-$	33	721	19	nd
	notch3$^+$	246	1741	8	3
	notch3$^-$	26	51	60	nd
